# Identification of DEAD-Box RNA Helicase DDX41 as a Trafficking Protein That Involves in Multiple Innate Immune Signaling Pathways in a Zebrafish Model

**DOI:** 10.3389/fimmu.2018.01327

**Published:** 2018-06-11

**Authors:** Jun-xia Ma, Jiang-yuan Li, Dong-dong Fan, Wei Feng, Ai-fu Lin, Li-xin Xiang, Jian-zhong Shao

**Affiliations:** ^1^College of Life Sciences, Key Laboratory for Cell and Gene Engineering of Zhejiang Province, Zhejiang University, Hangzhou, China; ^2^Laboratory for Marine Biology and Biotechnology, Qingdao National Laboratory for Marine Science and Technology, Qingdao, China

**Keywords:** zebrafish, DDX41, stimulator of IFN genes protein, signal transduction and activator of transcription 6, nuclear factor-κB, interferon, CCL20, innate immune signaling

## Abstract

DDX41 is an important sensor for host recognition of DNA viruses and initiation of nuclear factor-κB (NF-κB) and IFN signaling pathways in mammals. However, its occurrence and functions in other vertebrates remain poorly defined. Here, a DDX41 ortholog [*Danio rerio* DDX41 (*Dr*DDX41)] with various conserved structural features to its mammalian counterparts was identified from a zebrafish model. This *Dr*DDX41 was found to be a trafficking protein distributed in the nucleus of resting cells but transported into the cytoplasm under DNA stimulation. Two nuclear localization signal motifs were localized beside the coiled-coil domain, whereas one nuclear export signal motif existed in the DEADc domain. *Dr*DDX41 acts as an initiator for the activation of NF-κB and IFN signaling pathways in a *Danio rerio* STING (*Dr*STING)-dependent manner through its DEADc domain, which is a typical performance of mammalian DDX41. These observations suggested the conservation of DDX41 proteins throughout the vertebrate evolution, making zebrafish an alternative model in understanding DDX41-mediated immunology. With this model system, we found that *Dr*DDX41 contributes to *Dr*STING–*Danio rerio* STAT6 (*Dr*STAT6)-mediated chemokine (*Danio rerio* CCL20) production through its DEADc domain. To the best of our knowledge, this work is the first report showing that DDX41 is an upstream initiator in this newly identified signaling pathway. The *Dr*DDX41-mediated signaling pathways play important roles in innate antibacterial immunity because knockdown of either *Dr*DDX41 or *Dr*STING/*Dr*STAT6 significantly reduced the survival of zebrafish under *Aeromonas hydrophilia* or *Edwardsiella tarda* infection. Our findings would enrich the current knowledge of DDX41-mediated immunology and the evolutionary history of the DDX41 family.

## Introduction

The innate immune system detects pathogen-associated nucleic acids (bacterial or viral DNAs and RNAs) *via* pattern-recognition receptors (PRRs) to initiate host defense against pathogen infection by activating various innate immune signaling pathways that produce proinflammatory cytokines and IFNs (1). To date, numerous PRRs have been identified, which include the toll-like receptors (TLRs), the retinoic acid-inducible gene I (RIG-I)-like receptors, the nucleotide oligomerization domain-like receptors (NLRs), the C-type lectin receptors, and the family of cytosolic DNA sensors, such as DNA-dependent activator of IFN-regulatory factors (DAI), absent in melanoma 2 (AIM2), IFNγ-inducible protein 16 (IFI16), cyclic GMP-AMP synthase (cGAS), and DEAD (Asp-Glu-Ala-Asp) box polypeptide 41 (DDX41) (2–9). Among those receptors, the DDX41 has attracted considerable attention as a newly characterized cytosolic DNA sensor involving in a variety of innate immune reactions and the occurrence of some diseases (10). DDX41 dysfunction leads to many refractory disorders, such as the myelodysplastic syndrome and the acute myeloid leukemia (11).

DDX41 is a member of the DExD/H-box helicases superfamily. It was originally identified by its effect on axon outgrowth and fasciculation of the Bolwig nerve in the *Drosophila* called Abstrakt (12, 13). In an RNAi screen for 59 members of the superfamily, DDX41 was identified as a critical cytosolic DNA sensor mediated by the adaptor stimulator of IFN genes protein (STING) in a mouse DC line (D2SC cell line) (8). Structurally, DDX41 was characterized by two conserved RecA-like globular domains (DEADc and HELICc) containing various functional motifs which involve in ATP binding, ATP hydrolysis, nucleic acid recognition, and RNA unwinding (13). Functionally, DDX41 was ubiquitously expressed in various cells (such as dendritic cells and macrophages) to detect the foreign or endogenous double-stranded DNAs (dsDNAs) and bacterial cyclic dinucleotides (CDNs) such as cyclic di-AMP and cyclic di-GMP (9). DDX41 elicits nuclear factor-κB (NF-κB) and IFN signaling pathways by associating with STING through its DEADc domain to activate IκB kinase (IKK)β and TANK-binding kinase 1 (TBK1). Those activated kinases phosphorylate IκB and interferon regulatory factor 3/7 (IRF-3/7) to trigger the production of proinflammatory cytokines and IFNs (9). The function of DDX41 was regulated by E3 ubiquitin ligase TRIM21 and Bruton’s tyrosine kinase (BTK) through interfering DNA recognition and STING recruitment, respectively (14, 15). Given STING is the only downstream platform for DDX41, other STING-mediated signaling pathways, except for the currently known NF-κB and IFN ones, would also be the potential candidates initiated by the upstream DDX41. In fact, it has been recently found that the cytoplasmic nucleic acids can trigger STING to activate signal transduction and activator of transcription 6 (STAT6) and then induce the expression of the target genes like CCL2 and CCL20 which recruit immune cells to combat viral infection (16). Thus, whether DDX41 acts as the upstream sensor for this STING–STAT6-mediated signaling pathway becomes intriguingly to be elucidated. Clarification on this notion would broaden the current knowledge on the functional roles of DDX41 in innate immunity.

To date, knowledge on DDX41 was acquired mainly from human and mouse models. However, little is known about its occurrence and existence in other organisms. To deeply uncover the biological functions of DDX41 in innate immunity, different research models, particularly lower vertebrates, such as teleost fish, which possesses a well-established and complicated innate immune system, are being developed. Such nonmammalian research models would be greatly complementary to mammalian models to shed light on innate immunity and beneficial in providing a cross-species understanding of the evolutionary history of the DDX41 family throughout vertebrate evolution. Emerging evidence has shown that zebrafish (*Danio rerio*) is an attractive model organism for the study of comparative immunology and diseases. Actually, the major innate immune signaling pathways (such as NF-κB and IFN ones) and the pathway components, including the key PRRs (such as TLRs, NLRs, and RIG-I), adaptor proteins (such as myeloid differentiation primary-response protein 88 (MyD88), TIR-domain-containing adaptor protein inducing IFNβ (TRIF), and STING), regulatory factors (such as TRIM25 and suppressor of cytokine signaling (SOCS)), kinase molecules (such as TBK1 and IKKs), transcriptional factors (such as NF-κB, IRF3/7, and STAT1/6), and proinflammatory cytokines (such as IL-1β, IL-6, and TNFα), IFN and interferon-stimulated genes (ISGs) (such as IFNφ1/2/3/4, ISG15, and viperin), and chemokines (such as CCL20, CCL21, and CCL25) involving in the signaling pathways, have been characterized to be well conserved and orchestrated in zebrafish and other fish species (17–39). In addition, the STING molecule, as a pivotal adaptor protein connecting different innate signaling pathways (such as cGAS, IFI16, and DDX41), was also recently identified from zebrafish and other teleost fish, exhibiting high identity with that of the mammalian ones (5–9, 40). Therefore, zebrafish is expected to be a powerful tool for exploring DDX41-mediated immune activities.

In this study, we characterized a DDX41 ortholog [*Danio rerio* DDX41 (*Dr*DDX41)] from a zebrafish model. This *Dr*DDX41 shared highly conserved structural domains with its mammalian counterparts and was found to be a nuclear-localized protein capable of transferring into the cytoplasm in response to DNA stimulation. The nuclear localization signal (NLS) and nuclear export signal (NES) motifs were identified to exist beside the coiled-coil (CC) domain and in the DEADc domain, respectively. Functionally, *Dr*DDX41 can dramatically activate NF-κB and IFN signaling pathways in both zebrafish embryos and human HEK293T cells, leading to the upregulation of proinflammatory cytokines (TNFα and IL-6), type I IFNs, and ISGs (ISG15 and viperin). The results indicated the functional conservation of DDX41 family members in the innate immune signaling pathways between teleost fish and mammals, allowing a cross-species research for *Dr*DDX41 to be performed in mammalian models. By this strategy, we found that *Dr*DDX41 can act as an upstream mediator for the initiation of STING–STAT6-mediated CCL20 induction both in zebrafish and in HEK293/293T cells. This finding uncovered a previously unknown function of DDX41 in innate immune activities and indicated the wide participation of DDX41 in multiple innate immune reactions, adding a new functional performance to DDX41 family.

## Materials and Methods

### Experimental Fish

WT AB zebrafish, weighing ~1 g with ~2 cm body length, were kept in an aerated recirculating water system at 28°C under standard laboratory conditions as previously described (41). The fish were fed with commercial pellets twice a day. Only healthy fish, as determined by the general appearance and level of activity, were used in the experiments. Zebrafish embryos were acquired by natural spawning and kept at 28.5°C in incubator with a 14:10-h light/dark photoperiod (42). All animal care and experimental procedures were approved by the Committee on Animal Care and Use and the Committee on the Ethic of Animal Experiments of Zhejiang University.

### Molecular Cloning

The Genome and EST databases maintained by the NCBI, Ensembl, and DFCI were used to predict DDX41 ortholog in zebrafish by using mammalian DDX41 as queries, aided by GENSCAN, SMART, and BLAST software programs as previously described (43). Total RNA was isolated from kidney and spleen tissues by using an RNAiso Plus kit (Takara Bio), and *Dr*DDX41 cDNAs was generated through RT-PCR by using primers (as shown in Table S1 in Supplementary Material) in accordance with the manufacturer’s instructions (21). The PCR products were purified from agarose gel (1.2%) by using a gel extraction kit (Qiagen), inserted into the pGEM-T EASY vector (Promega), and then transformed into competent *Escherichia coli* DH5α (Invitrogen). The positive plasmid DNA was purified following the Miniprep protocol (Omega Bio-tek) and sequenced on an ABI 3730xl sequencer (Invitrogen) as previously described (44).

### Bioinformatics Analysis

Full-length *Dr*DDX41 cDNA was assembled using the CAP3 Sequence Assembly Program. Genome assemblies and locations were retrieved from the UCSC genome bioinformatics website and map viewer in the NCBI. Gene organization (intron/exon boundaries) was elucidated by comparing *Dr*DDX41 cDNA with genome sequence, and figures were drawn using GeneMapper 2.5. Multiple sequences alignments were generated using the ClustalW program (version 5.1), and the percentage of amino acid sequence identity was calculated using the MEGALIGN program from DNASTAR. Functional motifs in *Dr*DDX41 and other DDX41 proteins were analyzed using the SMART (45), cNLS mapper (46), UbPred program (47), and the Swiss Model (48). Tertiary structures of the functional domains were analyzed using SMART, SWISS-MODEL, and I-TASSER (49). Phylogenies of the protein sequences were estimated with MEGA5 software using the neighbor-joining method (50).

### Plasmid Construction

For functional analysis, the coding sequences (CDSs) for the full-length and various domain/residue-deleted mutant *Dr*DDX41 proteins were inserted into pCMV-Tag2B (Invitrogen) between BamHI/XhoI sites to construct eukaryotic expression vectors, including pCMV-Tag2B-*Dr*DDX41 (full-length), pCMV-Tag2B-*Dr*DDX41-ΔN (177–613 amino acids, lacking N-terminal region), pCMV-Tag2B-*Dr*DDX41-(ΔN + ΔDEADc) (412–613 amino acids, lacking N-terminal region and DEADc domain), pCMV-Tag2B-*Dr*DDX41-(ΔC + ΔHELICc) (1–411 amino acids, lacking C-terminal region and HELICc domain), and pCMV-Tag2B-*Dr*DDX41-ΔC (1–558 amino acids, lacking C-terminal region). The *Danio rerio* STING (*Dr*STING) expression construct (pCMV-N-Myc-*Dr*STING; Beyotime) was generated from an original plasmid (pcDNA1.1-*Dr*STING) kindly donated by Biacchesi (19). For subcellular localization or coimmunoprecipitation (Co-IP) analysis, the CDSs for the full-length and NLS-deleted or NES-mutated *Dr*DDX41 proteins or *Danio rerio* STAT6 (*Dr*STAT6) protein were inserted into pEGFP-N1 (Clontech) between XhoI/BamHI sites to construct EGFP-*Dr*DDX41 or EGFP-*Dr*STAT6 fusion protein expression vectors, including pEGFP-N1-*Dr*DDX41 (full-length), pEGFP-N1-*Dr*DDX41-ΔN (191–613 amino acids, lacking the N-terminal region), pEGFP-N1-*Dr*DDX41-N (1–190 amino acids, N-terminal region containing two NLS motifs), pEGFP-N1-*Dr*DDX41-DEADc (191–411 amino acids, DEADc domain), pEGFP-N1-*Dr*DDX41-NES-M (L322A and M326A point mutations in NES motif), and pEGFP-N1-*Dr*STAT6. The zebrafish IFN (*Dr*IFN) signaling pathway luciferase reporter vectors, including *Dr*IFNφ-1 promoter-luciferase reporter vector (*Dr*IFNφ-1-Luc) and *Dr*IFNφ-3 promoter-luciferase reporter vector (*Dr*IFNφ-3-Luc), were constructed according to the sequences as previously described (23). The human NF-κB, IRF3, and IFN-β luciferase reporter constructs and pRL-TK renilla luciferase reporter vector were purchased from Clontech and Promega, respectively. All primers used in plasmid construction are shown in Table S1 in Supplementary Material. The plasmids for transfection and microinjection were prepared endotoxin-free using an EZNA™ Plasmid Midi Kit (Omega Bio-Tek).

### Subcellular Localization and Trafficking Analysis

HEK293T cells were seeded onto cover slips in a 12-well plate and cultured in DMEM (Corning) supplemented with 10% (v/v) FCS (Gibco, Life Technologies), penicillin (100 U/mL), and streptomycin (100 µg/mL) at 37°C in 5% CO_2_. After growth until 70–90% confluence, cells were transfected with EGFP-tagged *Dr*DDX41/mutants expression vectors with a polyethyleneimine reagent (PEI, Sigma-Aldrich) according to the manufacturer’s instruction. After 24 h, cells were washed twice with PBS, fixed by 4% paraformaldehyde for 10 min, and stained with 100 ng/mL DAPI (Sigma-Aldrich) and 10 µM DiI (Beyotime) for 5–8 min at 37°C. Fluorescence images were obtained under an LSM 710 two-photon laser-scanning microscope (Zeiss, Jena, Germany) at 630× magnification. For trafficking analysis, cells were stimulated with poly(dA:dT) (1 µg/mL) and collected at 2 hours post stimulation (hps) and 6 hps for image examination.

### Immunofluorescence Staining

The distribution of *Dr*DDX41 in white blood cells (WBCs) was examined *via* immunofluorescence staining. For this, a rabbit anti-*Dr*DDX41 Ab was prepared from the peptide KDVLVATDVASKGLDFC, and followed by the protocols previously reported (51, 52). WBCs were separated from zebrafish peripheral blood, kidney, and spleen by Ficoll-Hypaque (1.080 g/mL) centrifugation (53, 54). Then, the cells were fixed with 4% paraformaldehyde for 10 min, blocked with 2% BSA (with 0.5% Triton-X100) for 2 h, and incubated with rabbit anti-*Dr*DDX41 at 4°C overnight. After washing with PBS, the cells were incubated with FITC-conjugated anti-rabbit Ab (Santa Cruz Biotechnology) for 40 min and DAPI (100 ng/mL) (Sigma-Aldrich) for 5 min. Imaging was performed under an LSM 710 laser-scanning microscope as described above.

### Morpholino (MO) and mRNA Synthesis

Morpholinos against *Dr*DDX41, *Dr*STING, and *Dr*STAT6 targeting the start codon regions and a nonspecific control MO were all ordered from Gene Tools and dissolved with nuclease-free H_2_O to 1 mM as stock solutions. The sequences of these MOs were listed in Table S1 in Supplementary Material. For mRNA synthesis, *Dr*DDX41, *Dr*STING, and *Dr*STAT6 full-length cDNA sequences were cloned into the pcDNA3.1 (Invitrogen) vector bewteen BamHI/XhoI sites using the primers shown in Table S1 in Supplementary Material. Capped mRNA was synthesized using the mMESSAGE kit (Ambion) and purified with Mini Quick Spin RNA columns (Roche) according to the manufacturer’s instructions.

### *In Vitro* Luciferase Reporter Assays

*In vitro* luciferase reporter assays were performed to examine *Dr*DDX41-mediated activation of NF-κB and IFN signaling pathways in HEK293T cells. For this, HEK293T cells were cultured in a 12-well plate and transfected with 15 ng renilla luciferase reporter vector and 150 ng NF-κB-Luc, or *Homo sapiens* IRF3-Luc (*Hs*IRF3-Luc), or *Hs*IFN-β-Luc reporter vector in combination with increasing amounts (0, 200, and 400 ng) of the expression vectors pCMV-Tag2B-*Dr*DDX41, pCMV-N-Myc-*Dr*STING individually or 200 ng pCMV-N-Myc-*Dr*STING plus increasing amounts (0, 200, and 400 ng) of pCMV-Tag2B-*Dr*DDX41. Empty control vector was added so that a total of 800 ng vector DNA was transfected into each well. At 24 h after transfection, cells were stimulated with PBS or poly(dA:dT) (1 µg/mL) delivered by PEI. Cells were collected after 6 h of stimulation. Luciferase activity in total cell lysates was detected with a Dual Luciferase Reporter Assay Kit (Promega) according to the manufacturer’s instruction.

### *In Vivo* Luciferase Reporter Assays

*In vivo* luciferase reporter assays were performed to examine *Dr*DDX41-mediated activation of NF-κB and IFN signaling pathways in zebrafish embryos through overexpression, knockdown, and rescue experiments. For overexpression experiment, the one-cell-stage embryo was microinjected (2 nL) with PBS/poly(dA:dT) (100 pg), 100 pg NF-κB-Luc or *Danio rerio* IFNφ1 (*Dr*IFNφ1)/3-Luc reporter vector, and 10 pg renilla luciferase reporter vector with increasing amounts (0, 50, 100, and 200 pg) of expression vectors pCMV-Tag2B-*Dr*DDX41, pCMV-N-Myc-*Dr*STING individually or 50 pg pCMV-N-Myc-*Dr*STING expression vector plus increasing amounts (0, 50, 100, and 200 pg) of pCMV-Tag2B-*Dr*DDX41. Empty control vector was added so that a total of 360 pg vector DNA was microinjected into each embryo. For knockdown and rescue experiments, the one-cell-stage embryo was microinjected with *Dr*DDX41/*Dr*STING-MO (4 ng), with/without *Dr*DDX41/*Dr*STING-mRNA (200 pg), in combination with poly(dA:dT), NF-κB-Luc/*Dr*IFNφ1/3-Luc reporter vector, and renilla luciferase reporter vector with same dosages as described above. The poly(dA:dT) and various plasmid DNAs were mixed in a microinjection buffer (0.5% phenol red, 240 mM KCl, and 40 mM HEPES, pH 7.4). Luciferase activity in total embryo lysates was detected with a Dual Luciferase Reporter Assay (Promega) at 24 hours post microinjection (hpm).

### Quantitative RT-PCR (Q-RT-PCR) for Expression Analysis

The expression of *Danio rerio* IL-6 (*Dr*IL-6), *Danio rerio* TNFα (*Dr*TNFα), *Dr*IFNφ1, *Danio rerio* Viperin (*Dr*Viperin), *Danio rerio* ISG15 (*Dr*ISG15), and CCL20 in response to *Dr*DDX41-initiated signaling pathways in HEK293T/HEK293 cells and zebrafish embryos was determined by Q-RT-PCR. For *Dr*IL-6, *Dr*TNFα, *Dr*IFNφ1, *Dr*Viperin, and *Dr*ISG15 analysis, HEK293T cells and embryos were administered with poly(dA:dT), MOs, mRNAs, and various expression vectors in different combinations as described in above luciferase reporter assays. For *Dr*STAT6-mediated CCL20 analysis, HEK293 cells were transfected with increasing amounts (0, 200, and 400 ng) of expression vectors for *Dr*DDX41, *Dr*STING, *Dr*STAT6 individually or 200 ng *Dr*STAT6 expression vector plus increasing amounts (0, 200, and 400 ng) of expression vector for *Dr*DDX41 or *Dr*STING. Empty control vector was added so that a total of 800 ng vector DNA was transfected into each well. Then, cells were stimulated with PBS or poly(dA:dT) and collected for *Hs*CCL20 expression analysis followed by the protocols and time intervals as described above. Besides, the one-cell-stage embryo was microinjected with poly(dA:dT) (100 pg) plus *Dr*DDX41-MO or *Dr*STING-MO or *Dr*STAT6-MO (4 ng) and *Dr*DDX41-mRNA or *Dr*STING-mRNA or *Dr*STAT6-mRNA (200 pg) in indicated combinations as follows: standard-MO, *Dr*DDX41-MO, *Dr*DDX41-MO + *Dr*DDX41-mRNA, *Dr*STING-MO, *Dr*STING-MO + *Dr*STING-mRNA, *Dr*STAT6-MO, *Dr*STAT6-MO + *Dr*STAT6-mRNA, *Dr*DDX41-MO + *Dr*STING-MO, *Dr*DDX41-MO + *Dr*DDX41-mRNA + *Dr*STING-MO, *Dr*DDX41-MO + *Dr*STING-MO + *Dr*STING-mRNA, *Dr*DDX41-MO + *Dr*DDX41-mRNA + *Dr*STING-MO + *Dr*STING-mRNA; the MO and mRNA for *Dr*DDX41 and *Dr*STAT6 as well as *Dr*STING and *Dr*STAT6 were in the same combination as for *Dr*DDX41 and *Dr*STING described above. Embryos were collected after 24 hpm. Total RNA was extracted from cells or 15–30 embryos using TRIzol reagent (Invitrogen). The transcripts of indicated molecules were analyzed *via* Q-RT-PCR on a Mastercycler ep realplex instrument (Eppendorf) (55). The relative expression levels were calculated using the 2^−ΔCt^ method with β-actin for normalization; and fold change was normalized by control to an arbitrary value of one. In all cases, the sample was run in triplicate parallel reactions. The experiments were repeated independently at least three times. The forward and reverse primers used were shown as in Table S1 in Supplementary Material.

### Co-IP and Western Blotting

The interaction between *Dr*DDX41 and *Dr*STING or *Dr*STING and *Dr*STAT6 was analyzed by Co-IP assay. For this, HEK293T cells were plated in 10-cm dishes (Corning) and transfected with 24 µL of PEI containing a total of 6 µg plasmid DNAs, including pCMV-Tag2B-*Dr*DDX41 or pCMV-Tag2B-*Dr*DDX41-mutants plus pCMV-N-Myc-*Dr*STING (at a ratio of 1:1); pCMV-N-Myc-*Dr*STING and pEGFP-N1-*Dr*STAT6 plus pCMV-Tag 2B-*Dr*DDX41 (at a ratio of 1:1:1), with the empty vector as control. After 24 h, the cells were stimulated with poly(dA:dT) (1 µg/mL) for 6 h, washed with PBS and lysed for 30 min at 4°C in an ice-cold buffer containing 20 mM Tris–HCl (pH 7.5), 150 mM NaCl, 1 mM EDTA, 1% Triton X-100, and the cocktail protease inhibitor (Roche). Cell lysates were centrifuged at 13,000 *g* for 15 min and the supernatants were incubated with mouse anti-Myc tag mAb (Abmart; M20002M) at 4°C overnight, followed by incubation with 50 µL protein A-agarose beads (Roche) for 3 h. Then, the beads were washed four times with lysis buffer; and the obtained protein samples were separated by 12% SDS-PAGE and transferred onto 0.45 µm polyvinylidene difluoride membranes (Bio-Rad). The blots were probed with mouse/rabbit anti-Myc/Flag/GFP tag mAb (Abmart; M20008M, M20004M, P30008M) at 1:5,000 and HRP-conjugated goat anti-mouse/rabbit IgG (Abmart; M21001S, M21002S) at 1:8,000, and then incubated with ECL reagents (Millipore) according to the manufacturer’s protocols. The emitted light was detected using a cooled CCD camera (LAS-1000; Fuji film).

### Challenge Experiment

The function of *Dr*DDX41-mediated signaling pathways in innate immunity was evaluated by its antibacterial activity in zebrafish embryo model. For this, the *Dr*DDX41/*Dr*STING/*Dr*STAT6 knockdown, rescue, and control embryos were prepared as described above; and these embryos were challenged with *A. hydrophila* (BSK-10) and *Edwardsiella tarda* (TL5m) (10^6^ cfu/mL), at 24 hours post fertilization (hpf) for 5 h as previously described (56, 57). The two virulent pathogens of infectious species in fish were provided by the Zhejiang Institute of Freshwater Fisheries. The mortality of each group was recorded at 12, 24, 36, 48, and 72 hours post infection, and the relative survival rate (RSR) was analyzed. RSR (%) = the survival rate of the infected group/the survival rate of the PBS-administered group.

### Statistical Analysis

Quantitative data are presented as the mean ± SD of each group. Statistical evaluation of differences between means of experimental groups was performed using Student’s *t*-tests. Statistical significance was considered at *p* < 0.05 or *p* < 0.01. All experiments were replicated at least three times.

## Results

### Identification of *Dr*DDX41 Gene

With human (*Homo sapiens*) DDX41 (*Hs*DDX41) gene sequence as a query, a homologous *Dr*DDX41 gene (GenBank accession number XM_017354423.1) was predicted from the zebrafish genome database. Similar to the existence of genes around the DDX41 locus on human chromosome 5, the HIGD2A, FAF2, SNCB, PDLIM7, N4BP3, and RMND5B homolog genes were all clustered around the *Dr*DDX41 gene on zebrafish chromosome 14, although the synteny of HIGD2A, N4BP3, and RMND5B gene loci was in a reverse order (Figure S1A in Supplementary Material). The organization of the *Dr*DDX41 gene was elucidated by comparing the *Dr*DDX41 cDNA with the corresponding genomic sequence. The *Dr*DDX41 gene is comprised of 17 exons and 16 introns, and located within a 26.2-kb genomic fragment on chromosome 14. This organization pattern of *Dr*DDX41 gene was similar to that of humans and other fish species predicted, such as olive flounder (*Paralichthys olivaceus*), medaka (*Oryzias latipes*), fugu (*Takifugu rubripes*), tetraodon (*Tetraodon nigroviridis*), and stickleback (*Gasterosteus aculeatus*) (data not shown). The exons 1–5, exons 6–11, exons 12–15, and exons 16 and 17 in the *Dr*DDX41 gene were predicted to encode the CC, DEADc, HELICc, and ZnF_C2HC domains, respectively, which were also consistent with each other among those of *H. sapiens* (Figure S1B in Supplementary Material). The cloned *Dr*DDX41 cDNA consists of 2,139 bp with a 107-bp 5′UTR, a 1,842-bp ORF, and a 191-bp 3′UTR containing a typical polyadenylation signal (AATAAA) nucleotide (Figure S2 in Supplementary Material).

### Structural Characterization of *Dr*DDX41 Protein

**Figure 1:**
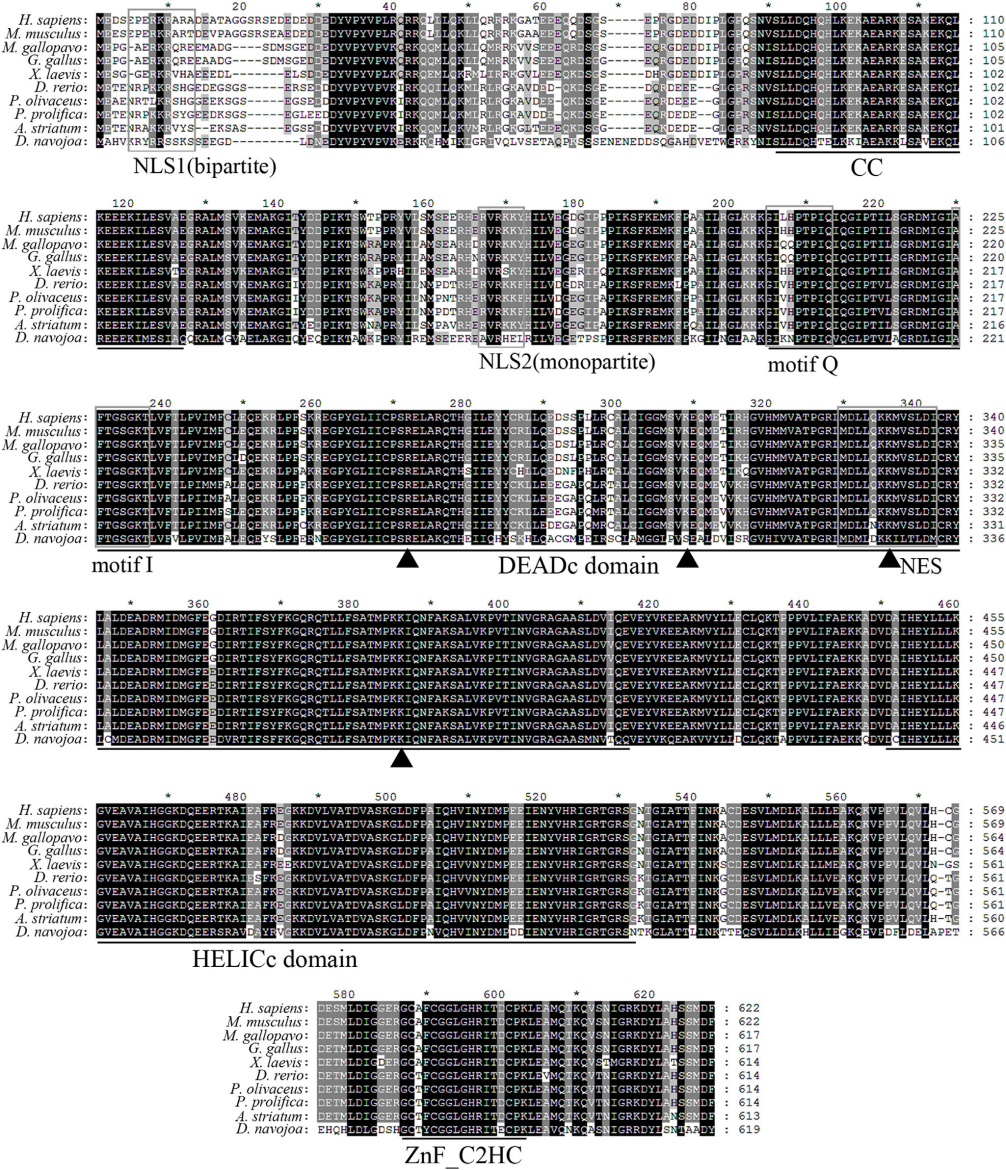
Multiple alignment of *Danio rerio* DDX41 with other orthologs. Residues shaded in black are completely conserved across all the species aligned, whereas residues shaded in gray are similar with respect to side chains. The dashes in the amino acid sequences indicate gaps introduced to maximize alignment. The four conserved domains coiled-coil (CC), DEADc, HELICc, and ZnF_C2HC are indicated below the alignment. The nuclear localization signal (NLS)1, NLS2, motif Q, motif I, and nuclear export signal (NES) consensus sequences are boxed. Triangles indicate the four important residues in the double-stranded DNA or cyclic dinucleotide binding.

*Danio rerio* DDX41 was predicted to be an intracellular protein without signal peptide and transmembrane domain, having an estimated molecular weight of 68.73 kDa and a theoretical isoelectric point of 8.08. Multiple sequence alignment analysis reveals that *Dr*DDX41 contains the conserved functional domains or motifs and shares 82–85% identity with that of mammalian counterparts and 88–92% identity with those of other fish species, such as salmon (*Salmon salar*) and olive flounder (*P. olivaceus*). It implies high conservation of DDX41 proteins among various species throughout the vertebrate evolution (Figure 1). Phylogenetic analysis showed that *Dr*DDX41 was clustered with other DDX41s of fish to form an exclusive group, which was merged with mammalian DDX41s into a large group with high bootstrap probability. The cluster of DDX41 in the phylogenetic tree was independent of other DDX41 family members, including DDX3, DDX23, DDX42, and DDX53 (Figure S3 in Supplementary Material). By SMART and I-TASSER analysis, the *Dr*DDX41 protein contains a CC domain, a DEADc domain, a HELICc domain, and a ZnF_C2HC domain, which is consistent with the domain architecture of *Hs*DDX41. The CC, DEADc, HELICc, and ZnF_C2HC domains of *Dr*DDX41 share an overall high amino acid identity of 85–90, 85–95, 85–95, and 79–92% with that of humans and other mammalian species, respectively. It suggests their importance in functional activities. According to the cNLS Mapper prediction and a previously reported judgment for NES, two NLS motifs (one monopartite and one bipartite) exist in the N-terminal region and one NES motif in the DEADc domain of *Dr*DDX41 (Figure 1; Figures S4A,B in Supplementary Material) (58). By UbPred program, a Lys106 residue was predicted in *Dr*DDX41, whose counterpart in *Hs*DDX41 (Lys115) was ubiquitinated by one of the E3 ubiquitin ligase (TRIM21) for the negative regulation of DDX41 *via* proteosomal degradation after the STING pathway has been triggered. In addition, the tyrosine phosphorylation site (Tyr414) in *Hs*DDX41, which was phosphorylated by BTK for the activation of DDX41 for DNA sensing, is also conserved in *Dr*DDX41 (Tyr405) (Figures S4A,B in Supplementary Material).

**Figure 2:**
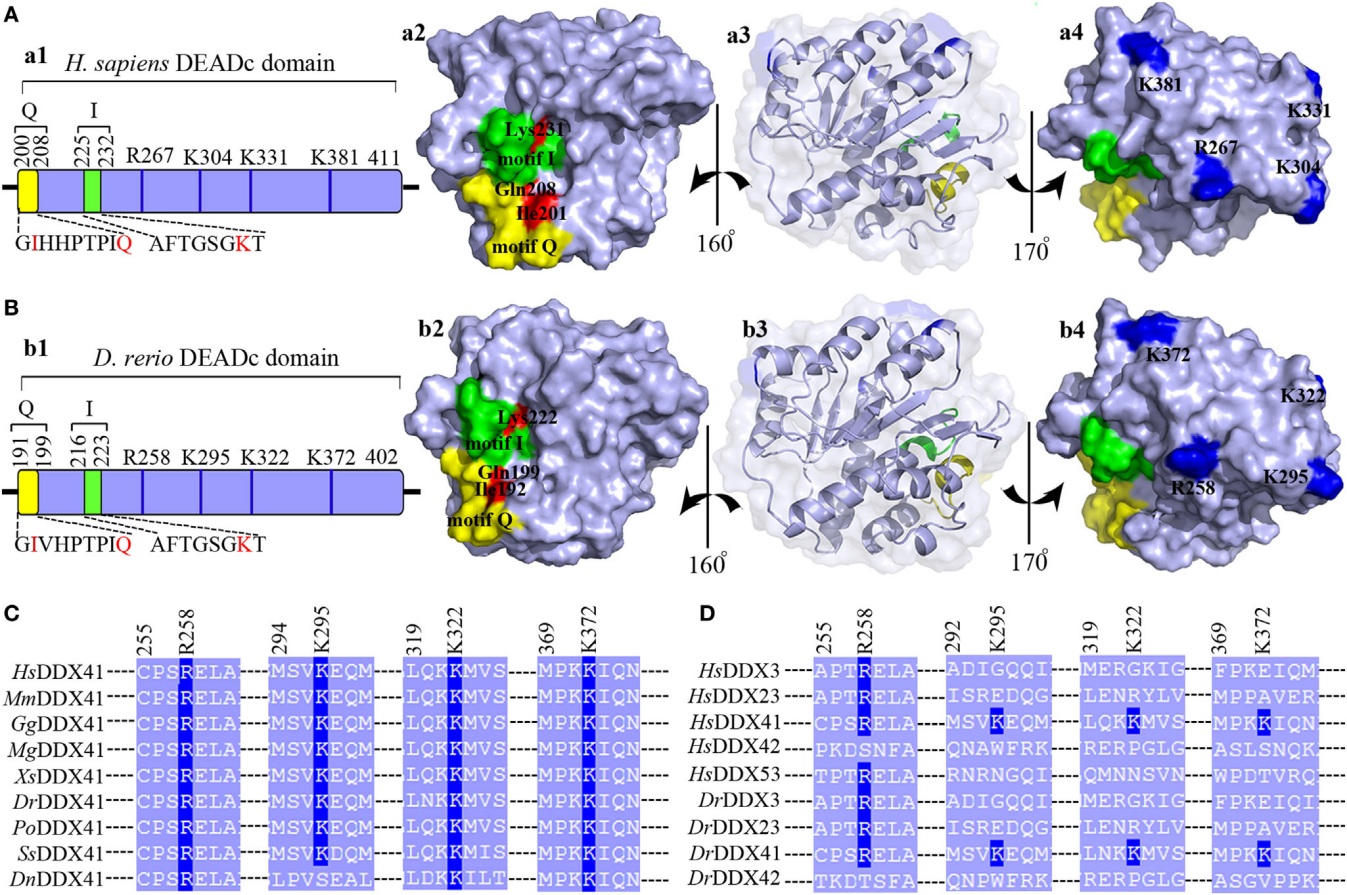
Motif organization of the DEADc domain. Schematic diagrams and tertiary structures of the functional motifs in DEADc domains of humans [**(A)**, in an APO open form; PDB ID: 5GVS] and zebrafish **(B)** were presented. (a1,b1) DEADc domain of humans (a1) and zebrafish (b1) contains a motif Q (yellow) and a motif I (green) at its N-terminal region and the key arginine (R) and lysine (K) residues (deep blue) at its C-terminal region (light blue). The color codes representing the motifs and the N- or C-terminal regions are used similarly in the other figures, unless otherwise stated. (a2,b2) Molecular surface representation of the residues involving in recognizing the adenine moiety and the β- and γ-phosphate groups of ATP in *Homo sapiens* DDX41 (*Hs*DDX41) (a2) and *Danio rerio* DDX41 (*Dr*DDX41) (b2) in motif Q and motif I. (a3,b3) Molecular surface and α-helixes and β-sheets representation of DEADc domain in a same view with Figures S4C,D (lower). (a4,b4) Molecular surface representation of the residues involving in the putative double-stranded DNA (dsDNA)/cyclic dinucleotide (CDN) binding (colored in deep blue). **(C)** Amino acid sequence of the residues near the putative dsDNA/CDN-binding sites of *Dr*DDX41 and its orthologs **(C)**, as well as its paralogs **(D)**. Residues critical for dsDNA or CDN binding are indicated in deep blue. The accession numbers for the sequences of DDX41 and its orthologs and paralogs are shown in Figure S3.

Given the central roles of DEADc domain in the functional activity of DDX41, this domain was selected for further comparative analysis. The tertiary structure of the *Dr*DDX41 DEADc domain was modeled by using the crystal structure of *Hs*DDX41 DEADc domain (PDB ID: 5H1Y) as a template (59). Results showed that the *Dr*DDX41 DEADc domain clearly adopts a similar RecA-like fold seen in *Hs*DDX41 DEADc domain, which containing one core β-sheets surrounded by 9 α-helixes. Particularly, the C-terminal regions (224–390 amino acids) have almost the same conformation between the *Dr*DDX41 and *Hs*DDX41 DEADc domain (Figures S4C,D in Supplementary Material). Furthermore, the Ile192 and Gln199 (corresponding to Ile201 and Gln208 in *Hs*DDX41), two important residues in the *Hs*DDX41 motif Q crucial for recognizing the adenine moiety in a base-specific manner; and the main chain amide groups together with Lys222 (corresponding to Lys231 in *Hs*DDX41), which was believed to extensively recognize the β- and γ-phosphate groups of ATP in *Hs*DDX41 motif I, were completely conserved in the *Dr*DDX41 DEADc domain, respectively (Figure 2A, a1–a3; Figure 2B, b1–b3). The putative dsDNA binding site located around the surface of the C-terminal region of the DEADc domain, and the key amino acid residues Arg258, Lys295, and Lys372 (corresponding to Arg267, Lys304, and Lys381 in *Hs*DDX41) involving in the dsDNA and CDN recognition were typical in *Dr*DDX41 DEADc domain. Besides, the Lys322 residue (corresponding to Lys331 in *Hs*DDX41) exclusively involving in CDN binding was also detected in *Dr*DDX41 DEADc domain (Figure 2A, a1–a4; Figure 2B, b1–b4). These results suggested that the dsDNA- and CDN-binding sites of *Dr*DDX41 overlap with each other as they were in *Hs*DDX41. Notably, the Lys295, Lys322, and Lys372 residues (corresponding to Lys304, Lys331, and Lys381 in *Hs*DDX41) on the dsDNA- and CDN-binding surface are highly conserved in the orthologs of DDX41 from other species (Figure 2C), while they were not conserved in other paralogs of DDX41 family, such as DDX3, DDX23, DDX42, and DDX53 (Figure 2D). These observations are consistent with the fact that the dsDNA- and CDN-binding activities are specific features of DDX41.

### Tissue Distribution and Embryo Expression of *Dr*DDX41

**Figure 3:**
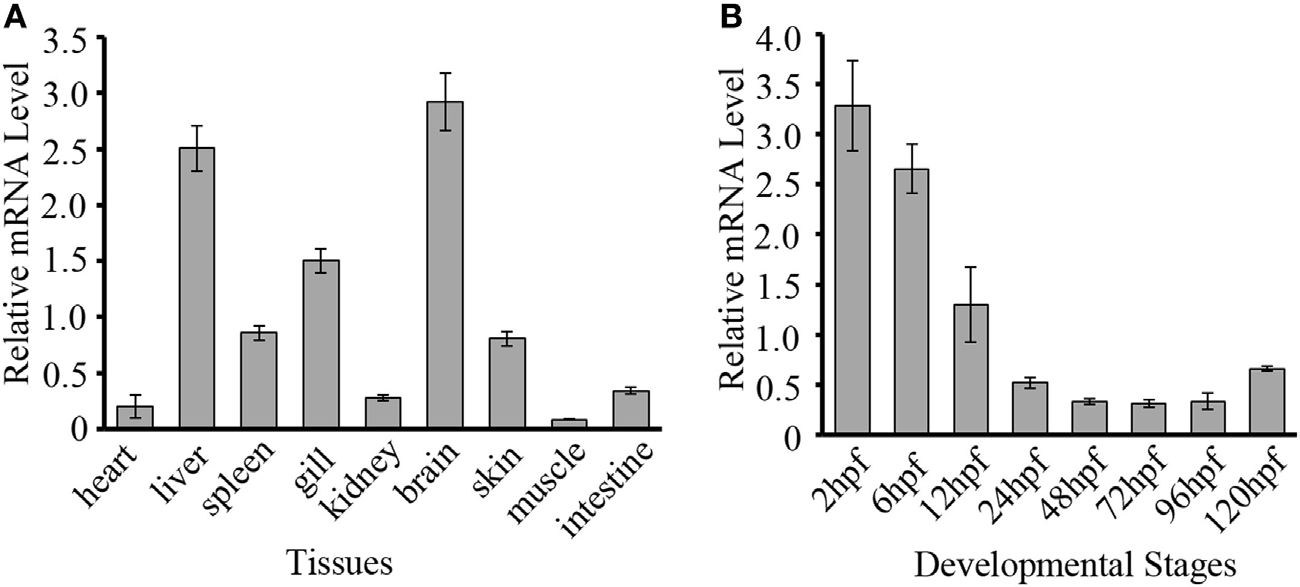
Expression patterns of *Danio rerio* DDX41 (*Dr*DDX41) gene in different tissues and embryos at different developmental stages. **(A)** Expression profiles of *Dr*DDX41 mRNA in the different tissues as indicated. **(B)** Expression profiles of *Dr*DDX41 gene in embryos at the different developmental stages [2–120 hours post fertilization (hpf)]. β-actin was chosen as the internal control for normalization. Relative expression data were calculated by the method of 2^−ΔCt^. The *vertical bars* represent the mean ± SD (*n* = 3). The data are from three independent experiments performed in triplicate.

To evaluate the potential role of *Dr*DDX41 in zebrafish and the usage of zebrafish embryo model for functional study, the expression profiles of *Dr*DDX41 transcripts were analyzed in various tissues of adult zebrafish and in early developmental stages of embryos. Results showed that *Dr*DDX41 was widely expressed in a range of tissues examined, including heart, liver, spleen, gill, kidney, brain, skin, muscle and intestine (Figure 3A). Specifically, *Dr*DDX41 mRNA was highly expressed in brain and immune-relevant tissues, including liver, gill, spleen, and skin, implying the wide participation of *Dr*DDX41 in various physiological and immunological activities. In addition, *Dr*DDX41 was also found to be constitutively expressed in zebrafish embryo from 2 to 120 hpf, with the highest level at 2–6 hpf and a moderate level at 12 hpf (Figure 3B).

### Subcellular Localization of *Dr*DDX41

**Figure 4:**
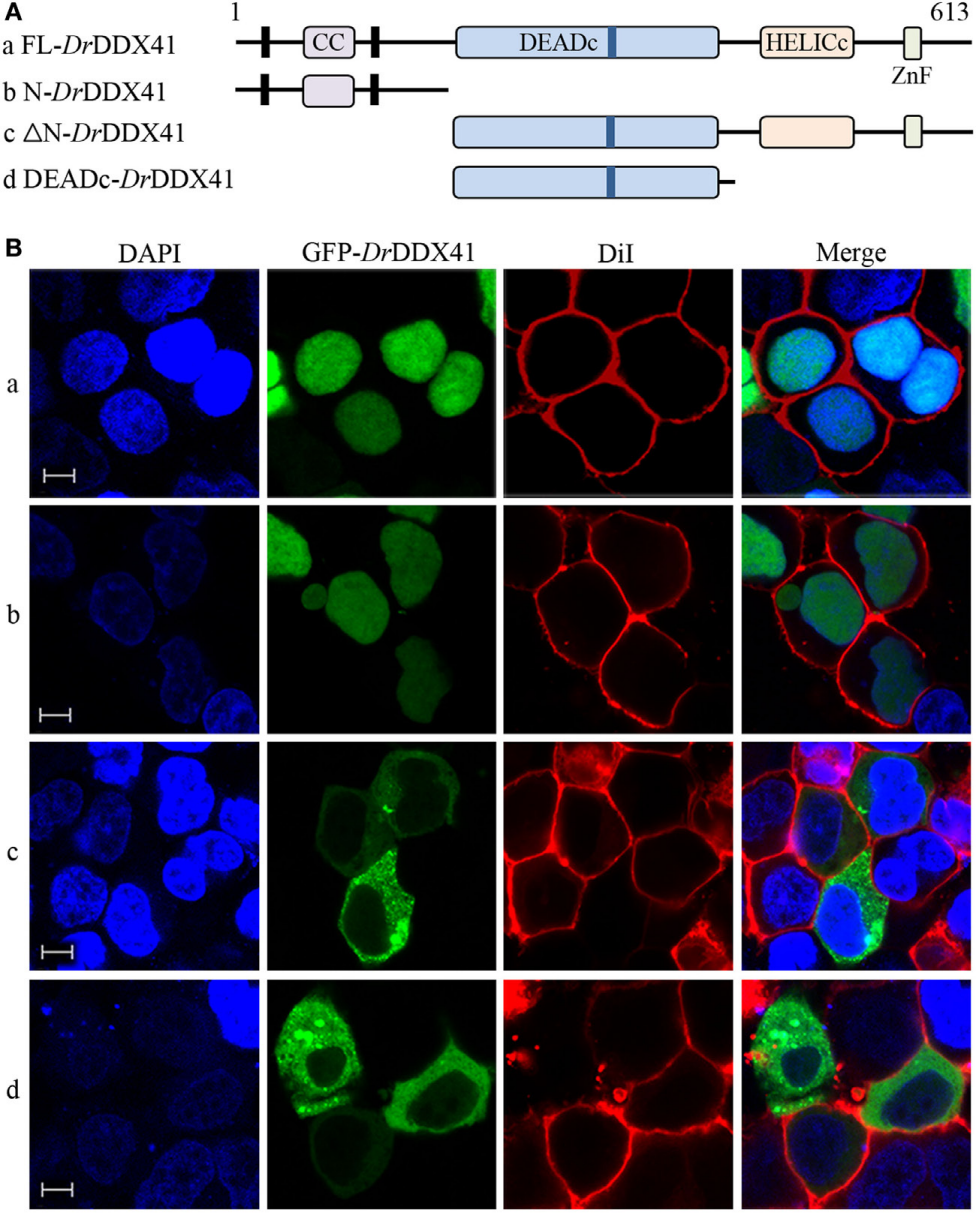
Subcellular localization of *Danio rerio* DDX41 (*Dr*DDX41) and identification of the nuclear localization signal motifs within it. **(A)** Schematic diagram of full-length (FL) and *Dr*DDX41 mutants with the coiled-coil (CC), DEADc, HELICc, ZnF_C2HC domains, and residue numbers as indicated. The various *Dr*DDX41 fragments were inserted into the C-terminus of pEGFP-N1. **(B)** Representative images of transfected HEK293T cells. The N-terminal 190 amino acids targeted GFP to the nucleus (a,b). Deletion of this region in *Dr*DDX41 (191–613, c) and *Dr*DDX41 (191–411, d) resulting in punctate cytoplasmic staining and exclusion from the nucleus. Scale bars represent 5 µm. Images were captured under a laser-scanning confocal microscope (Zeiss LSM-710; original magnification, 630×).

**Figure 5:**
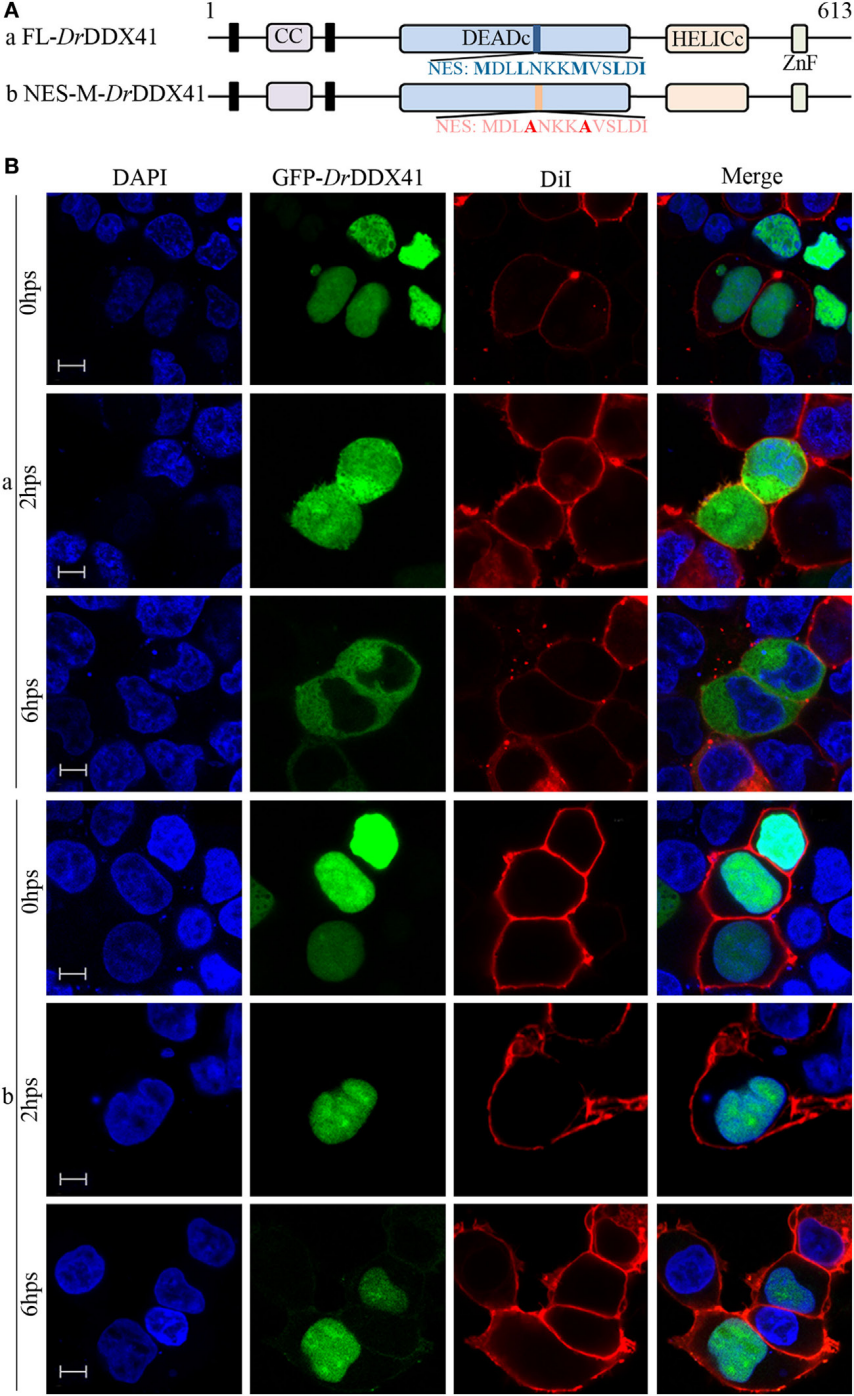
Trafficking analysis of *Danio rerio* DDX41 (*Dr*DDX41) and identification of the nuclear export signal (NES) motif within it. **(A)** Schematic diagram of full-length and the NES-mutated *Dr*DDX41. **(B)** Trafficking of *Dr*DDX41 in response to poly(dA:dT) stimulation at different hours post stimulation (hps) and the contribution of NES motif to the trafficking. Stimulation with poly(dA:dT) induced transportation of *Dr*DDX41 (full-length, a) from nucleus to cytoplasm. Meanwhile, the NES-like-mutant *Dr*DDX41 (NES-M, b) remained in the nucleus without any response to poly(dA:dT) stimulation. Scale bars represent 5 µm. Images were captured under a laser-scanning confocal microscope (Zeiss LSM-710; original magnification, 630×).

To investigate the subcellular localization of *Dr*DDX41 and whether the predicted NLS motifs worked, various EGFP-fused *Dr*DDX41 mutant protein-encoding plasmids were constructed (Figure 4A) and transfected into HEK293T cells. Results showed that the N-terminal region (1–190 amino acids) exhibited the same nuclear localization as the full-length WT *Dr*DDX41 protein did (Figure 4B, a,b). However, the mutant protein (191–613 amino acids) with the deletion of this N-terminal (1–190 amino acids) fragment in *Dr*DDX41 resulted in distinct punctate cytoplasmic distribution and loss of nuclear localization (Figure 4B, c). The mutant with only the DEADc domain in *Dr*DDX41 (191–411 amino acids) showed a similar cytosolic distribution pattern with no obvious nuclear localization (Figure 4B, d). These observations suggested that the N-terminal region of *Dr*DDX41 contains the main NLSs capable of targeting the protein into the nucleus, which is consistent with the prediction that two NLS motifs (NLS1: ENRAKKRVY and NLS2: ERVRKKYH) exist at the N-terminus. Considering that *Dr*DDX41 should be a cytosolic DNA sensor located in the cytoplasm, we suppose that *Dr*DDX41 could be transported from the nucleus into the cytoplasm under stimulation with certain DNA molecules. As expected, after stimulation with poly(dA:dT) (1 µg/mL) for 6 h, the majority of the *Dr*DDX41 signal was distributed in the cytoplasm (Figure 5B, a). To explore the NES motif that contributes to the transportation of *Dr*DDX41 from the nucleus, a potential NES-like sequence (NES: MDLLNKKMVSLDI; acting in a CRM1-dependent nuclear export pathway) was predicted, and a NES-like-mutated encoding plasmid was constructed (Figure 5A). Results showed that the NES-like-mutated *Dr*DDX41 was no longer transported from the nucleus, as determined by little distribution of this mutant could be detected in the cytoplasm under poly(dA:dT) stimulation (Figure 5B, b). Thus, *Dr*DDX41 may be predominantly preserved in the nucleus after synthesis and was recruited into the cytoplasm in response to the DNA stimuli. To further elucidate the subcellular localization of *Dr*DDX41 in zebrafish cells, the WBCs were sorted from the peritoneal blood, kidney, and spleen, and the immunofluorescence staining assay of *Dr*DDX41 was conducted using rabbit anti-*Dr*DDX41 antibody, whose specificity has been tested by western blot analysis as shown in Figure S5A in Supplementary Material. As expected, the nuclear distribution of *Dr*DDX41 can be detected in WBCs, and considerable amount of *Dr*DDX41 was also found in the cytoplasm (Figure S5B in Supplementary Material). In addition, we have also examined the subcellular localization of *Dr*DDX41 in zebrafish ZF4 cells, and a similar nuclear distribution was observed (Figure S5C in Supplementary Material). These observations suggested that a trafficking property of *Dr*DDX41 actually exists.

### Involvement of *Dr*DDX41 in NF-κB Signaling Pathway

**Figure 6:**
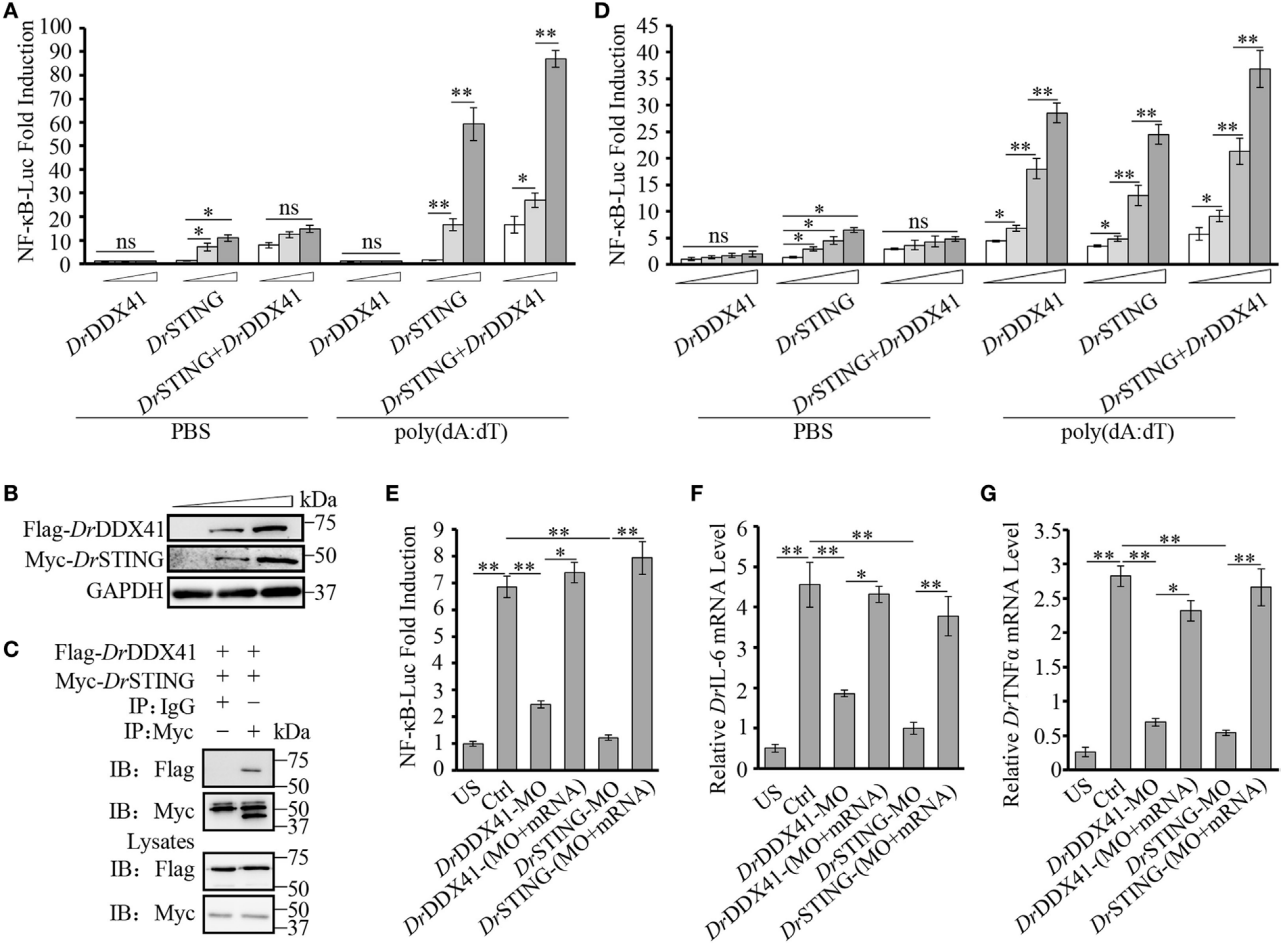
Involvement of *Danio rerio* DDX41 (*Dr*DDX41) in nuclear factor-κB (NF-κB) signaling pathway. **(A)** Activation of the NF-κB-binding promoter in HEK293T cells transfected with an NF-κB luciferase reporter (NF-κB-Luc; 150 ng/mL), a renilla luciferase reporter (15 ng/mL) plus increasing amounts (0, 200, and 400 ng/mL) of expression vectors for *Dr*DDX41 or *Danio rerio* STING (*Dr*STING) individually or expression vector for *Dr*STING (200 ng/mL) together with increasing amounts (0, 200, and 400 ng/mL) of expression vectors for *Dr*DDX41 and stimulated with poly(dA:dT) (1 µg/mL) for 6 h. Data are the average luciferase activity ± SD (*p < 0.05; **p < 0.01). ns, not significant. **(B)** Western blot analysis for the expression of *Dr*DDX41 and *Dr*STING in HEK293T cells transfected with increasing amounts (0, 200, and 400 ng/mL) of corresponding vectors. **(C)** *Dr*DDX41 interacts with *Dr*STING. HEK293T cells were transfected with Flag-*Dr*DDX41 and Myc-*Dr*STING for 24 h. Cell lysates were immunoprecipitated with anti-Myc antibody (Myc) or control mouse IgG (IgG), and analyzed by western blot using anti-Flag and anti-Myc antibodies. Expression of the transfected plasmids was analyzed with anti-Flag and anti-Myc antibodies in the whole cell lysates. **(D)** Activation of the NF-κB-binding promoters in embryos microinjected with an NF-κB luciferase reporter (NF-κB-Luc; 100 pg/embryo), a renilla luciferase reporter (10 pg/embryo), and poly(dA:dT) (100 pg/embryo) plus increasing amounts (0, 50, 100, and 200 pg/embryo) of expression vectors for *Dr*DDX41 or *Dr*STING individually or expression vector for *Dr*STING (50 pg/embryo) together with increasing amounts (0, 50, 100, and 200 pg/embryo) of expression vectors for *Dr*DDX41. Data are the average luciferase activity ± SD (*p < 0.05; **p < 0.01). **(E)** Activation of the NF-κB-binding promoter in embryos microinjected with an NF-κB luciferase reporter (NF-κB-Luc; 100 pg/embryo), a renilla luciferase reporter (10 pg/embryo) plus standard MO and PBS (US, unstimulated, administered with standard MO and PBS) or plus standard MO [Ctrl, administered with standard MO and poly(dA:dT)], *Dr*DDX41/*Dr*STING-MO, the targeted-MOs together with their mRNAs, and poly(dA:dT) (100 pg/embryo). Data are the average luciferase activity ± SD (*p < 0.05; **p < 0.01). **(F,G)** Quantitative RT-PCR analysis of *Danio rerio* IL-6 **(F)** and *Danio rerio* TNFα **(G)** mRNA levels in embryos microinjected with standard MO and PBS (US) or plus standard MO (Ctrl), *Dr*DDX41/*Dr*STING-MO, the targeted-MOs together with their mRNAs, and poly(dA:dT) (100 pg/embryo). Each of the MOs and mRNAs was administered at 4 ng/embryo and 200 pg/embryo in each group. Data are representative of three independent experiments as mean ± SD (*p < 0.05; **p < 0.01). Standard loading was indicated by β-actin expression.

Given the highly structural conservation between *Dr*DDX41/*Dr*STING and their mammalian counterparts, we suppose that the functional performance of *Dr*DDX41 and *Dr*STING in innate immune signaling pathways would be also conserved among different species, and thus the role of *Dr*DDX41/*Dr*STING could be examined (at least partly) in a mammalian cellular system. For this, the HEK293T cell, a widely used human model cell line naturally having minimal (or lacking) expression of DDX41 and STING proteins, was initially used in our study (16, 60). Transfection of HEK293T cells with different amounts of vectors for *Dr*DDX41 or *Dr*STING in different combination was conducted. As expected, overexpression of *Dr*STING in HEK293T cells dramatically induced (*p* < 0.01) the NF-κB reporter activation in a dose-dependent manner. By contrast, minimal NF-κB reporter activation could be detected in the cells only overexpressed with *Dr*DDX41. This result may be largely due to the lack of STING expression in HEK293T cells. Thus, a *Dr*STING compensation assay was performed by co-expression of *Dr*DDX41 and *Dr*STING in HEK293T cells and the functional association between *Dr*DDX41 and *Dr*STING was evaluated by a dose-dependent assay, wherein cells were administered with various amounts of *Dr*DDX41 in combination with a designated amount of *Dr*STING. Results showed that the activation of NF-κB was significantly induced (*p* < 0.05 or *p* < 0.01) in *Dr*DDX41 and *Dr*STING co-expressed cells, and the induction was enhanced in a *Dr*DDX41 dose-dependent manner (Figure 6A). Western blot analysis revealed that the expression of both *Dr*DDX41 and *Dr*STING can be detected under this circumstance (Figure 6B). To further investigate the relationship between *Dr*DDX41 and *Dr*STING, we investigated the potential interaction between them through overexpressing Flag-tagged *Dr*DDX41 and Myc-tagged *Dr*STING vectors in HEK293T cells and found that considerable *Dr*STING was precipitated by anti-Flag mAb (Figure 6C). These results suggested that *Dr*DDX41 involves in NF-κB signaling pathway by associating with *Dr*STING, which acts as a downstream mediator for *Dr*DDX41-initiated signaling as its orthologs did in mammalian systems.

To provide further support, *in vivo* functional characterization was performed by using zebrafish embryo as a model because it has a constitutive and moderate expression of *Dr*DDX41 and *Dr*STING during its early development (2–24 hpf). For this, an enhanced activation of NF-κB reporter was initially examined by administering the embryos with different amounts of vectors for *Dr*DDX41 or *Dr*STING or the combination of them for 24 h. Results showed that overexpression of *Dr*DDX41 and *Dr*STING (alone or in combination) significantly increased (*p* < 0.05 or *p* < 0.01) the NF-κB activation in a dose-dependent manner (Figure 6D). Then, the start-codon-targeted-MO oligonucleotide-based knockdown and MO-resistant-capped-mRNA-based rescue assays were conducted. The knockdown of either *Dr*DDX41 or *Dr*STING in embryos significantly inhibited the activation of NF-κB under poly(dA:dT) stimulation, and the impaired NF-κB activation was dramatically rescued (*p* < 0.05 or *p* < 0.01) by the MO-resistant *Dr*DDX41 and *Dr*STING mRNAs, respectively (Figure 6E). Correspondingly, the expression of *Dr*TNFα and *Dr*IL-6, two typical proinflammatory cytokines regulated by NF-κB signaling pathway, in *Dr*DDX41 and *Dr*STING morphants also significantly declined in response to poly(dA:dT) stimulation. Moreover, the impaired expression of these two cytokines was remarkably rescued by the administration of the MO-resistant *Dr*DDX41 and *Dr*STING mRNAs (Figures 6F,G).

### Involvement of *Dr*DDX41 in IFN Signaling Pathway

**Figure 7:**
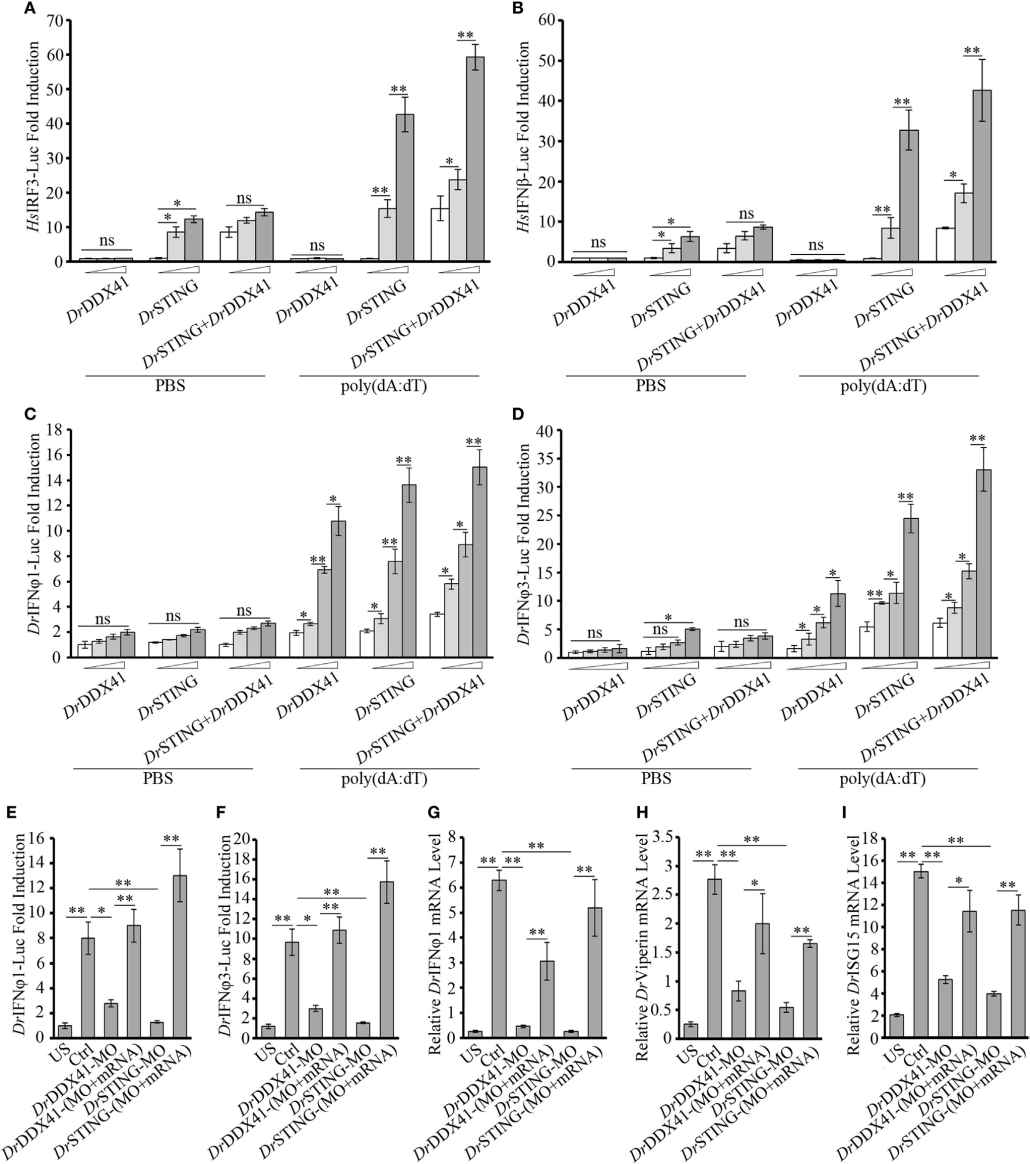
Involvement of *Danio rerio* DDX41 (*Dr*DDX41) in IFN signaling pathway. **(A,B)** Activation of the *Hs*IRF3 **(A)** or the *Hs*IFNβ **(B)** promoter in HEK293T cells transfected with an *Hs*IRF3 luciferase reporter [*Homo sapiens* IRF3-Luc (*Hs*IRF3-Luc); 150 ng/mL] or *Hs*IFNβ luciferase reporter [*Homo sapiens* IFNβ-Luc (*Hs*IFNβ-Luc); 150 ng/mL], and a renilla luciferase reporter (15 ng/mL) plus increasing amounts (0, 200, and 400 ng/mL) of expression vectors for *Dr*DDX41 or *Danio rerio* STING (*Dr*STING) individually or expression vectors for *Dr*STING (200 ng/mL) together with increasing amounts (0, 200, and 400 ng/mL) of expression vectors for *Dr*DDX41 and stimulated with poly(dA:dT) (1 µg/mL) for 6 h. Data are the average luciferase activity ± SD (*p < 0.05; **p < 0.01). **(C,D)** Activation of the *Danio rerio* IFNφ1 (*Dr*IFNφ1) **(C)** or the *Dr*IFNφ3 **(D)** promoter in embryos microinjected with an *Dr*IFNφ1 luciferase reporter (*Dr*IFNφ1-Luc; 100 pg/embryo) or *Dr*IFNφ3 luciferase reporter (*Dr*IFNφ3-Luc; 100 pg/embryo), a renilla luciferase reporter (10 pg/embryo), and poly(dA:dT) (100 pg/embryo) plus increasing amounts (0, 50, 100, and 200 pg/embryo) of expression vectors for *Dr*DDX41 or *Dr*STING individually or expression vector for *Dr*STING (50 pg/embryo) together with increasing amounts (0, 50, 100, and 200 pg/embryo) of expression vectors for *Dr*DDX41 at one-cell stage. Data are the average luciferase activity ± SD (*p < 0.05; **p < 0.01). **(E,F)** Activation of the *Dr*IFNφ1 **(E)** or the *Dr*IFNφ3 **(F)** promoter in embryos microinjected with an *Dr*IFNφ1 luciferase reporter (*Dr*IFNφ1-Luc; 100 pg/embryo) or *Dr*IFNφ3 luciferase reporter (*Dr*IFNφ3-Luc; 100 pg/embryo), a renilla luciferase reporter (10 pg/embryo) plus standard MO and PBS (US) or plus standard MO (Ctrl), *Dr*DDX41/*Dr*STING-MO, the targeted-MOs together with their mRNAs, and poly(dA:dT) (100 pg/embryo). Data are the average luciferase activity ± SD (*p < 0.05; **p < 0.01). **(G–I)** Quantitative RT-PCR analysis of *Dr*IFNφ1 **(G)**, *Danio rerio* Viperin **(H)** and *Danio rerio* ISG15 **(I)** mRNA levels in embryos microinjected with standard MO and PBS (US) or plus standard MO (Ctrl), *Dr*DDX41/*Dr*STING-MO, the targeted-MOs together with their mRNAs, and poly(dA:dT) (100 pg/embryo). Each of the MOs and mRNAs was administered at 4 ng/embryo and 200 pg/embryo in each group. Data are representative of three independent experiments as mean ± SD (*p < 0.05; **p < 0.01). Standard loading was indicated by β-actin expression.

As shown in NF-κB investigation, the HEK293T cell line is an attractive model system for the cross-species study of *Dr*DDX41–*Dr*STING-mediated innate immune signaling pathways. Thus, the involvement of *Dr*DDX41/*Dr*STING in IFN signaling pathway was also initially evaluated in HEK293T cells. Similarly, overexpression of *Dr*STING in cells by transfecting with various amounts of vector for *Dr*STING dramatically induced the activation of human IRF3/IFNβ (*Hs*IFRF3/IFNβ) reporters in a dose-dependent manner, and minimal *Hs*IFRF3/IFNβ reporters activities were detected in the cells only expressed with *Dr*DDX41. However, when *Dr*DDX41 was co-expressed with *Dr*STING in cells, the activation of *Hs*IRF3/IFNβ reporters was significantly restored (*p* < 0.01), and the restoration was in a *Dr*DDX41 dose-dependent manner (Figures 7A,B). These results suggested that *Dr*DDX41 also involves in IFN signaling by association with *Dr*STING; and the performance of DDX41/STING in IFN signaling pathway was conserved from fish to humans.

Next, *in vivo* involvement of *Dr*DDX41/*Dr*STING in IFN signaling was characterized in zebrafish embryos by using two luciferase reporters linked to *Dr*IFNφ1 and *Dr*IFNφ3 (two typical type I interferon molecules in zebrafish) promoters, respectively. Results showed that overexpression of *Dr*DDX41 or *Dr*STING in embryos by microinjecting with various amounts of the two expression vectors significantly activated (*p* < 0.05) the *Dr*IFNφ1 and *Dr*IFNφ3 luciferase reporters; and the activation was enhanced with the increasing capacity of the expression vectors administered (Figures 7C,D). By MO-based knockdown and mRNA-based rescue assays, it was found that ablation of *Dr*DDX41 and *Dr*STING in embryos significantly inhibited the *Dr*IFNφ1/3 reporter activity in response to poly(dA:dT) stimulation, and the impaired *Dr*IFNφ1/3 reporter activation could be significantly stored by the MO-resistant mRNAs for *Dr*DDX41 and *Dr*STING (Figures 7E,F). Consequently, the expression of *Dr*Viperin, *Dr*ISG15, and *Dr*IFNφ1 in *Dr*DDX41- and *Dr*STING-knockdown morphants also significantly declined in response to poly(dA:dT) stimulation; and the impaired expression of these genes was significantly rescued (*p* < 0.01) by the administration of the MO-resistant mRNAs (Figures 7G–I).

### Involvement of *Dr*DDX41 in *Dr*STING-*Dr*STAT6-Mediated *Danio rerio* CCL20 (*Dr*CCL20) Expression

**Figure 8:**
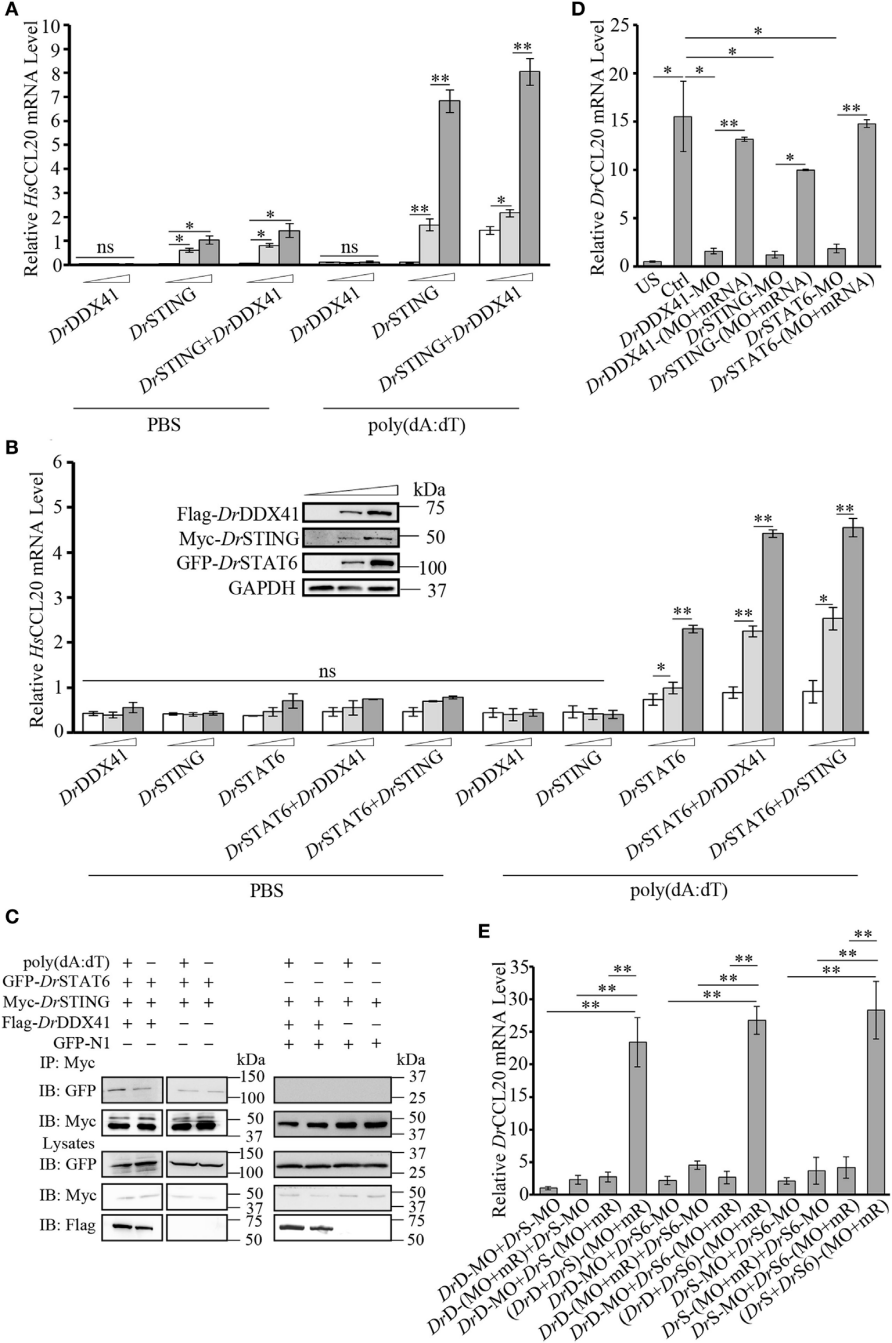
Involvement of *Danio rerio* DDX41 (*Dr*DDX41) in *Danio rerio* STING (*Dr*STING)–*Danio rerio* STAT6 (*Dr*STAT6)-mediated *Danio rerio* CCL20 (*Dr*CCL20) expression. **(A)** Quantitative RT-PCR (Q-RT-PCR) analysis of *Hs*CCL20 in HEK293T cells transfected with increasing amounts (0, 200, and 400 ng/mL) of expression vectors for *Dr*DDX41 or *Dr*STING individually or expression vector for *Dr*STING (200 ng/mL) together with increasing concentrations (0, 200, and 400 ng/mL) of expression vectors for *Dr*DDX41 and stimulated with poly(dA:dT) (1 µg/mL) for 6 h. Data are representative of three independent experiments as mean ± SD (*p < 0.05; **p < 0.01). **(B)** Q-RT-PCR analysis of *Hs*CCL20 in HEK293 cells transfected with increasing amounts (0, 200, and 400 ng/mL) of expression vectors for *Dr*DDX41, *Dr*STING or *Dr*STAT6 individually or expression vector for *Dr*STAT6 (200 ng/mL) together with increasing concentrations (0, 200, and 400 ng/mL) of expression vectors for *Dr*DDX41 or *Dr*STING and stimulated with poly(dA:dT) (1 µg/mL) for 6 h. Data are representative of three independent experiments as mean ± SD (*p < 0.05; **p < 0.01). The bands in the upper-left denote the western blot analysis of the expression of *Dr*DDX41, *Dr*STING, and *Dr*STAT6 in HEK293 cells transfected with increasing amounts (0, 200, and 400 ng/mL) of corresponding vectors. **(C)** *Dr*STING coimmunoprecipitates with *Dr*STAT6 through the mediation of *Dr*DDX41 under poly(dA:dT) stimulation. **(D)** Q-RT-PCR analysis of *Dr*CCL20 mRNA levels in 24 hours post microinjection embryos microinjected with standard MO and PBS (US) or standard MO (Ctrl), *Dr*DDX41/*Dr*STING/*Dr*STAT6-MO, the targeted-MOs together with their corresponding mRNAs, and poly(dA:dT) (100 pg/embryo). Data are representative of three independent experiments as mean ± SD (*p < 0.05; **p < 0.01). Standard loading was indicated by β-actin expression. **(E)** Q-RT-PCR analysis of *Dr*CCL20 mRNA levels in embryos microinjected with different combinations of *Dr*DDX41-MO (*Dr*D-MO), *Dr*STING-MO (*Dr*S-MO), *Dr*STAT6-MO (*Dr*S6-MO), and their mRNAs (mR) for rescue, and poly(dA:dT) (100 pg/embryo). Each of the MOs and mRNAs was administered at 4 ng/embryo and 200 pg/embryo in each group. Data are representative of three independent experiments as mean ± SD (*p < 0.05; **p < 0.01). Standard loading was indicated by β-actin expression.

Given the observation that the activation of STAT6 by STING is critical for CCL20 expression in antiviral innate immunity in mammals, the involvement of *Dr*DDX41 in STING–STAT6-mediated CCL20 expression was investigated. For this, the HEK293T (lacking/having a low expression of STING and DDX41) and the HEK293 (lacking a functional endogenous STAT6 but expressing STING and DDX41) cell lines were selectively used. Results showed that overexpression of *Dr*STING or *Dr*DDX41 combined with *Dr*STING in HEK293T cells induced the robust expression of human (*H. sapiens*) CCL20 (*Hs*CCL20) in a *Dr*STING or *Dr*DDX41 dose-dependent manner (Figure 8A). However, overexpression of *Dr*DDX41 and *Dr*STING in HEK293 cells induced minimal expression of *Hs*CCL20; and the expression of *Hs*CCL20 was induced only under the ectopic introduction of *Dr*STAT6 into the cells. These results suggest that the *Dr*DDX41–*Dr*STING-mediated CCL20 expression was in a *Dr*STAT6-dependent manner. As expected, the *Hs*CCL20 mRNA level increased significantly (*p* < 0.01) in the cells administered with various amounts of vectors for *Dr*DDX41 or *Dr*STING in combination with a designated amount of *Dr*STAT6 (Figure 8B). Western blot analysis revealed that the expression of *Dr*DDX41, *Dr*STING, and *Dr*STAT6 can be detected under this circumstance (Figure 8B). To give further evidence, Co-IP assay was conducted in HEK293T cells overexpressed with Myc-tagged-*Dr*STING and GFP-tagged-*Dr*STAT6 together with/without Flag-tagged-*Dr*DDX41 to evaluate the interaction between *Dr*STING and *Dr*STAT6 under poly(dA:dT) stimulation. GFP and Myc-tagged-*Dr*STING expression vectors were transfected as control. Results showed that *Dr*STING clearly interacts with *Dr*STAT6 in *Dr*DDX41 co-expressed cells; and the interaction was significantly enhanced by the stimulation of poly(dA:dT), as determined by a stronger Co-IP signal in poly(dA:dT) stimulated cells than that of mock PBS treated ones. In contrast, little interaction was detected between *Dr*STING and *Dr*STAT6 in cells without *Dr*DDX41 expression (Figure 8C; Figure S6 in Supplementary Material).

By *in vivo* functional characterization in zebrafish embryo model, it was found that MO-based knockdown of *Dr*DDX41, *Dr*STING, and *Dr*STAT6 in embryos all significantly inhibited (*p* < 0.05) the induction of *Dr*CCL20 under poly (dA:dT) stimulation, and the impaired *Dr*CCL20 expression in these morphants was dramatically rescued (*p* < 0.01) by the MO-resistant mRNAs for *Dr*DDX41, *Dr*STING and *Dr*STAT6, respectively (Figure 8D). These results suggested the existence of a *Dr*DDX41–*Dr*STING–*Dr*STAT6-mediated signaling pathway for *Dr*CCL20 expression. To provide further support, a rescue assay was performed in morphants with double knockdown of *Dr*DDX41, *Dr*STING, and *Dr*STAT6 in different combinations. The results showed that the impaired *Dr*CCL20 expression in double morphants (*Dr*DDX41–*Dr*STING, *Dr*DDX41–*Dr*STAT6, or *Dr*STING–*Dr*STAT6) could be significantly restored (*p* < 0.01) only by the double administration of the two corresponding MO-resistant mRNAs, rather than by any single MO-resistant mRNA independently (Figure 8E). This observation provides functional correlation among *Dr*DDX41, *Dr*STING and *Dr*STAT6 in the signaling pathway.

### Function of Different *Dr*DDX41 Domains in the Signaling Pathways

**Figure 9:**
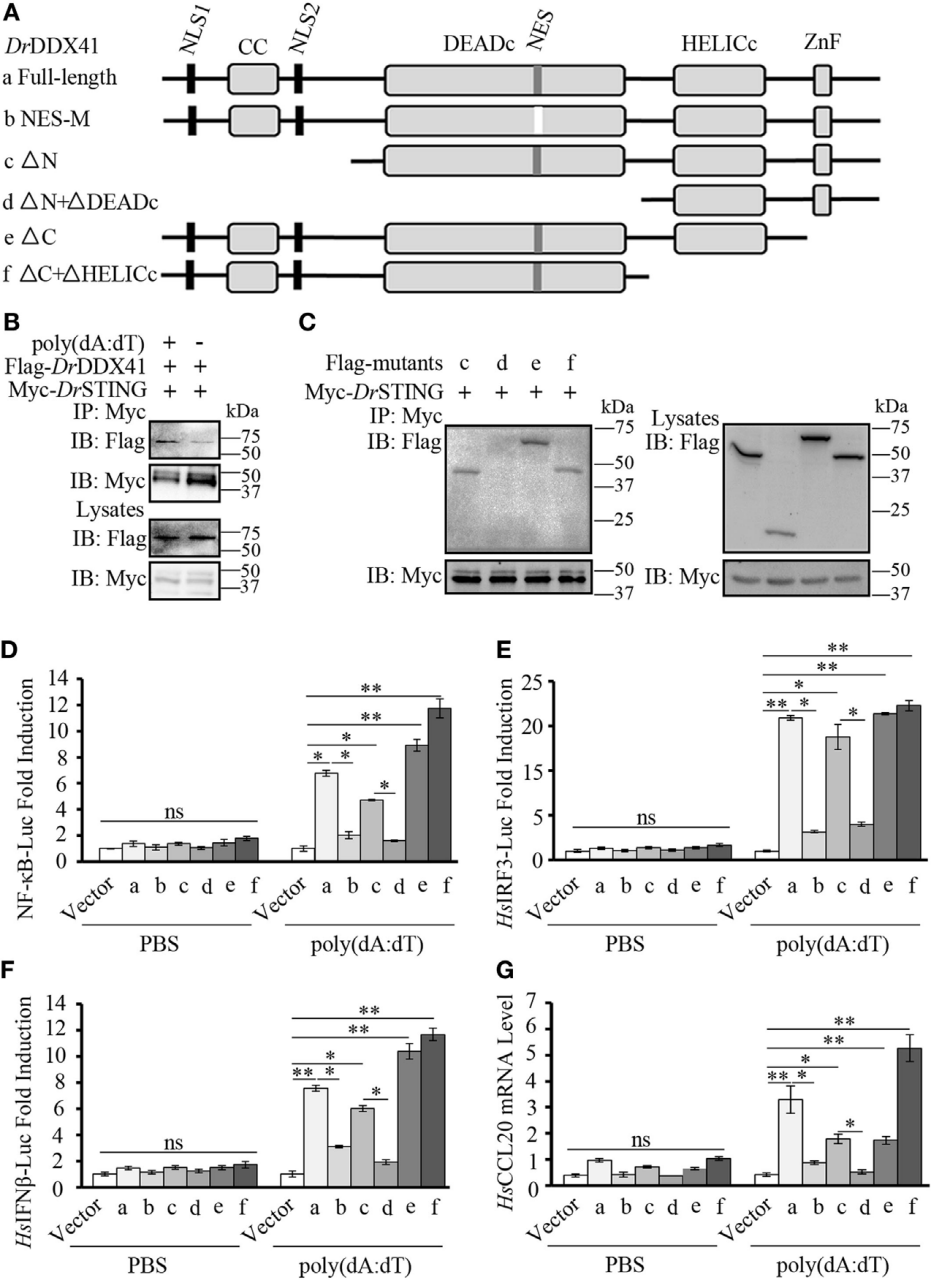
Function of different *Danio rerio* DDX41 (*Dr*DDX41) domains in the signaling pathways. **(A)** Schematic diagram of full-length and mutated *Dr*DDX41 fragments, which were inserted into the C-terminus of pCMV-Tag2B. **(B,C)** *Dr*DDX41 **(B)** and its mutants [**(C)**, constructed as in panel **(A)**] interact with *Danio rerio* STING (*Dr*STING). HEK293T cells were transfected with Flag-*Dr*DDX41/its mutants and Myc-*Dr*STING for 24 h. Cell lysates were immunoprecipitated with anti-Myc antibody (Myc) and analyzed by western blot using anti-Flag and anti-Myc antibodies. Expression of the transfected plasmids was analyzed with anti-Flag and anti-Myc antibodies in the whole cell lysates. **(D–F)** Activation of the nuclear factor-κB (NF-κB)-binding **(D)**, *Hs*IRF3 **(E)**, or *Hs*IFNβ **(F)** promoters in HEK293T cells transfected with an NF-κB/*Hs*IRF3/*Hs*IFNβ-Luc reporter (150 ng/mL) and a renilla luciferase reporter (15 ng/mL) plus different vectors of *Dr*DDX41 (400 ng/mL) together with pCMV-N-Myc-*Dr*STING (200 ng/mL) and stimulated with poly(dA:dT) (1 µg/mL) for 6 h. Data are the average luciferase activity ± SD (*p < 0.05; **p < 0.01). **(G)** Quantitative RT-PCR analysis of *Hs*CCL20 mRNA levels in HEK293T cells transfected with different constructions of *Dr*DDX41 (400 ng/mL) plus pCMV-N-Myc-*Dr*STING (200 ng/mL) and stimulated with poly(dA:dT) (1 µg/mL) for 6 h. Data are representative of three independent experiments as mean ± SD (*p < 0.05; **p < 0.01). Standard loading was indicated by β-actin expression.

To further validate the association of *Dr*DDX41 with *Dr*STING in the innate immune signaling pathways and the functional roles of different *Dr*DDX41 domains underlying this process, various *Dr*DDX41 mutants that lack the N-terminal region (177–613 amino acids, named as ΔN), the N-terminal region and DEADc domain (412–613 amino acids, named as ΔN + ΔDEADc), the C-terminal region (1–558 amino acids, named as ΔC), the C-terminal region and HELICc domain (1–411 amino acids, named as ΔC + ΔHELICc), and mutate at NES motif (named as NES-M) were constructed for Co-IP assay and functional evaluation (Figure 9A). It was showed that more Flag-tagged-*Dr*DDX41 was precipitated by anti-Myc mAb in HEK293T cells co-expressed with Myc-tagged-*Dr*STING after stimulation with poly(dA:dT) (Figure 9B). Moreover, clear Co-IP signals were observed from HEK293T cells co-expressed with *Dr*STING and the mutants of ΔN, ΔC + ΔHELICc, and ΔC, all of which contain the DEADc domain. However, no interaction could be detected between *Dr*STING and the ΔN + ΔDEADc mutant, the latter of which misses the DEADc domain (Figure 9C). Clearly, the DEADc domain contributes to the association of *Dr*DDX41 with *Dr*STING. Functional assays showed that overexpression of ΔN, ΔC + ΔHELICc, and ΔC mutant proteins in HEK293T cells significantly induced (*p* < 0.05 or *p* < 0.01) the NF-κB, *Hs*IRF3, and *Hs*IFNβ luciferase reporter activation and *Hs*CCL20 expression. Meanwhile, no significant activation of these reporters or *Hs*CCL20 expression could be detected in ΔN + ΔDEADc mutant protein overexpressed cells (Figures 9D–G). The results indicated that the DEADc domain of *Dr*DDX41 is required for the activation of *Dr*DDX41–*Dr*STING-mediated NF-κB, IFN, and CCL20 signaling pathways. This functional observation was in agreement with those from the interaction assay showing that DEADc domain contributes to the association of *Dr*DDX41 with *Dr*STING. Interestingly, the activation of NF-κB, *Hs*IRF3, and *Hs*IFNβ luciferase reporters or the expression of *Hs*CCL20 was much stronger in ΔC + ΔHELICc transfected cells than that in cells transfected with full-length *Dr*DDX41 or the ΔC (Figures 9D–G). This finding suggested that the HELICc domain probably causes some kind of obstruction of the signaling pathways. In addition, no significant activation of the NF-κB, *Hs*IRF3, and *Hs*IFNβ luciferase reporters or *Hs*CCL20 expression was detected in NES-mutant *Dr*DDX41 (NES-M) co-expressed cells (Figures 9D–G). This outcome was consistent with the observation that the NES-mutant *Dr*DDX41 could no longer be transported to the cytoplasm from the nucleus (Figure 5B, b).

### Functional Evaluation of *Dr*DDX41 in Innate Antibacterial Immunity

**Figure 10:**
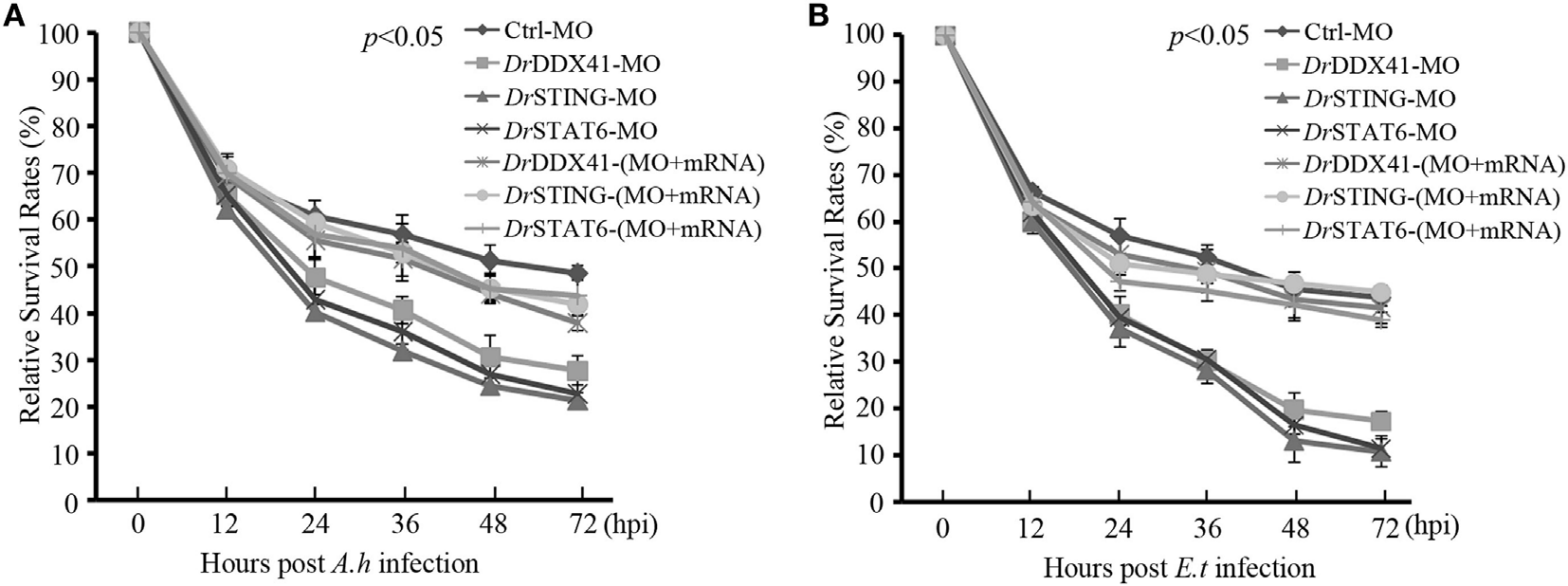
Antibacterial function of *Danio rerio* DDX41 (*Dr*DDX41) in zebrafish embryo model. Relative survival rates of *Aeromonas hydrophilia* **(A)** and *Edwardsiella tarda*-infected **(B)** zebrafish embryos in knockdown and rescue experiments. Zebrafish embryos were microinjected with standard-MO (Ctrl-MO), *Dr*DDX41/*Dr*STING/*Dr*STAT6-MO or together with their corresponding mRNAs, and raised to 24 hours post fertilization. Then, the embryos were exposed to PBS, *A. hydrophilia*, or *E. tarda* at 10^6^ cfu/mL for 5 h by immersion and monitored for 72 h. The survived embryos were calculated at 12, 24, 36, 48, and 72 hours post infection. Data are representative of three independent experiments as mean ± SD (*p < 0.05; **p < 0.01).

Functional role of *Dr*DDX41–*Dr*STING-mediated signaling pathways in innate antibacterial immunity was evaluated in zebrafish embryos (with knockdown of *Dr*DDX41, *Dr*STING, or *Dr*STAT6) under infection with *Aeromonas hydrophilia* (10^6^ cfu/mL) or *E. tarda* (10^6^ cfu/mL), two virulent pathogens for various fish species, for 3 days. Results showed that the mortality in *Dr*DDX41, *Dr*STING, and *Dr*STAT6 knockdown morphants challenged with *A. hydrophilia* (78 ± 2.53, 85 ± 1.21, and 83 ± 2.25%) and *E. tarda* (86 ± 2.66, 92 ± 1.50, and 91 ± 2.72%) was significantly (*p* < 0.05) higher than those of standard control morphants challenged with *A. hydrophilia* (44 ± 1.43%) and *E. tarda* (39 ± 1.77%). The survival of the *Dr*DDX41, *Dr*STING, and *Dr*STAT6 morphants could be rescued by the MO-resistant *Dr*DDX41, *Dr*STING, and *Dr*STAT6 mRNAs as determined by the significant decline of the mortality in *Dr*DDX41, *Dr*STING, and *Dr*STAT6-rescued morphants challenged with *A. hydrophilia* (66 ± 1.43, 62 ± 1.22, and 61 ± 3.84%) and *E. tarda* (64 ± 1.22, 62 ± 1.35, and 67 ± 4.27%). The RSR (%) of each experimental group was shown as in Figures 10A,B.

## Discussion

Although DDX41 has been well investigated in humans and mice, there are still few studies on this molecule in teleost fish (39, 61, 62). In this study, we described the molecular and functional characteristics of a DDX41 ortholog (*Dr*DDX41) from a zebrafish model. A number of conserved structural features among the DDX41 molecules of zebrafish and other species support the conclusion that the identified *Dr*DDX41 is a member of the DDX41 family. The evidence includes similar gene organization and chromosomal synteny, high amino acid sequence identity, and conserved tertiary structure, functional domains (such as CC, DEADc, HELICc, and ZnF_C2HC domains), motifs (such as motif Q and motif I), key amino residues, and modification (such as phosphorylation and ubiquitination) sites. The existence of high structural similarity between *Dr*DDX41 and its orthologs in humans and other mammalian species provides insights into the functional conservation of the DDX41 family members in innate immunity throughout the vertebrate evolution from teleost fish to mammals.

To provide experimental evidence for this notion, functional performance of *Dr*DDX41 in innate immune signaling pathways and responses was evaluated both *in vitro* and *in vivo*. For *in vitro* assay, the HEK293T and/or HEK293 cell line, which naturally has a low or lacks the expression of endogenous DDX41, STING, and STAT6 was used as a model system. By ectopic expression of *Dr*DDX41, *Dr*STING, and *Dr*STAT6 in HEK293T and/or HEK293 cells alone or in different combinations, *Dr*DDX41 can dramatically induce the activation of NF-κB and IFN signaling pathways and the expression of CCL20. The activity of the NF-κB-Luc, *Hs*IRF3-Luc, and *Homo sapiens* IFNβ-Luc reporters and the expression of CCL20 in the experimental cells were significantly upregulated in a dose-dependent manner along with the *Dr*DDX41. By Co-IP assay, strong interaction exists between *Dr*DDX41 and *Dr*STING or between *Dr*STING and *Dr*STAT6 during the activation of the signaling pathways. These results indicate that *Dr*DDX41 involves in NF-κB, IFN, and STAT6-mediated signaling pathways in a *Dr*STING-dependent manner. Given the fact that *Dr*DDX41 and *Dr*STING interaction can initiate the innate immune signaling pathways in a human cell line, it suggests that the key components and mechanisms underlying the DDX41-STING-mediated signaling pathways would be well conserved from teleost fish to humans. This conclusion could be supported by the recent identification of the major members of the signaling pathways downstream the STING, such as TBK1, IKKβ, IRF3/7, STAT6, and CCL20, from zebrafish and other fish species, all of which have high identities to their mammalian counterparts (23–39). The alternative use of human cell line (HEK293T or HEK293) for *Dr*DDX41 provides a cross-species research model, which is greatly beneficial in the understanding of *Dr*DDX41-mediated immune responses, considering there are not enough zebrafish cell lines available.

*In vivo* involvement of *Dr*DDX41 in *Dr*STING-mediated signaling pathways was investigated in zebrafish embryos. The zebrafish embryo is becoming an attractive model system for the investigation of innate immunity, because the majority of the innate immune system, including various innate immune cell lineages and molecules, are well developed and expressed in the embryo at early developmental stages (12–48 hpf); and the zebrafish embryo possesses many advantages, such as easy preparation and genetic manipulation (63). Consistent with the *in vitro* result, the *Dr*DDX41 and *Dr*STING clearly participated in the NF-κB, IFN, and STAT6-CCL20 signaling pathways *in vivo*. Knockdown of *Dr*DDX41 and *Dr*STING (in combination with or without knockdown of *Dr*STAT6) significantly inhibited the activation of NF-κB-Luc and *Dr*IFNφ1/3-Luc reporters and the expression of proinflammatory cytokines (*Dr*IL-6 and *Dr*TNFα), *Dr*IFNφ1, *Dr*ISGs (*Dr*Viperin and *Dr*ISG15), and *Dr*CCL20. These inhibitions can be substantially recovered by the administration of the MO-resistant mRNAs in morphants in the rescue assays.

It was well believed that STING is a central adaptor protein connecting various innate signaling pathways through association with IKKβ, TBK1, and MAVS by its conserved pLxIS motif (64). Aside from the involvement of STING in the classical NF-κB and IFN signaling pathways, it was recently discovered that STING also participates in STAT6-mediated chemokine CCL20 production, which could attract various immune cells, like CCR6-expressing B cell, T cells, and dendritic cells (16). However, the upstream receptor for the initiation of the STING-STAT6 signaling pathway remains to be clarified. In this study, we provide functional insights into the involvement of DDX41 in this signaling pathway. To the best of our knowledge, this is the first report showing the involvement of DDX41 in STAT6-mediated chemokine production. This finding suggests that DDX41 has a much wider range of effects on multiple innate signaling pathways and immunological activities than previously known. Given the high conservation of DDX41 in innate immunity across different organisms, the function of DDX41 in STING-STAT6-mediated chemokine production may be universally conserved in other species including humans. However, direct evidence is still needed to clarify this implication. Furthermore, it also remains to be elucidated whether other cytosolic receptors connecting the STING adaptor, such as cGAS and IFI16, participate in this STING-STAT6-mediated chemokine production. In addition, it is interesting to explore the question of under what conditions would *Dr*DDX41–*Dr*STING activate the NF-κB, IFN, and STAT6-mediated signaling pathways. In this respect, it was known that IKKβ is required for NF-κB activation, and TBK1 is responsible for the stimulation of IFN- and STAT6-mediated signaling pathways (6–9, 16, 25, 65, 66). The IKKβ and TBK1 were negatively regulated by NEMO and YAP/TAZ by formation of NEMO/IKKβ and YAP/TAZ/TBK1 inhibitory complexes and could be differentially activated by the ubiquitination of NEMO and phosphorylation of YAP/TAZ *via* TRIM32/TRIM56- and Lats1/2-dependent pathways under different cellular metabolic/nutrient stresses or bacterial/viral infections (65, 67). These observations suggest that TRIM32/TRIM56/NEMO/IKKβ and Lats1/2/YAP/TAZ/TBK1 axes downstream of *Dr*DDX41–*Dr*STING largely contribute to the differential activation of NF-κB, IFN, and STAT6-mediated signaling pathways, whose performance might be closely regulated by the cellular metabolic status in different cell types or the metabolic alteration in a certain cell type in response to bacterial/viral challenge. Moreover, TBK1 was also reciprocally required for NF-κB activation, probably by directly phosphorylating IKKβ (65, 66). This finding suggests the existence of a complex feedback/network regulatory mechanism underlying *Dr*DDX41–*Dr*STING-mediated signaling pathways. Further study is needed to clarify these questions, which depends on the fully understanding of the cellular distribution pattern of *Dr*DDX41–*Dr*STING signaling pathways, the metabolomics on pathogen infections, and even the correlation between *Dr*DDX41 and other cytosolic dsDNA sensors connecting the STING (such as cGAS and IFI16) as a whole.

In spite of the structural and functional conservation of DDX41, the subcellular localization of DDX41 seems elusive because it was initially found to be a cytosolic protein in HEK293T cells and D2SC cells (8, 9). However, several subsequent investigations have shown that DDX41 is located in the nucleus of HEK293, murine lung fibroblast, and THP1 cells (60, 68). DDX41 was also able to undergo a nucleocytoplasmic shuttling in CHO cells (69). Thus, a cytosolic DNA receptor being localized in the nucleus remains confusing. In this study, we found that *Dr*DDX41 mostly distributed in the nucleus in HEK293T cells without any stimulation. However, considerable *Dr*DDX41 proteins transport from the nucleus into the cytoplasm when cells were stimulated with poly(dA:dT). Similar results were also seen in zebrafish WBCs sorted from the peripheral blood, kidney, and spleen tissues. These observations suggested that DDX41 is a trafficking protein that is originally located in the nucleus of the resting cells but transported into the cytoplasm in response to DNA stimulation. This induced trafficking event might provide a regulatory strategy that allows DDX41-expressing cells to avoid unnecessary activation before the invasion of foreign DNA into the cytoplasm. Certainly, further study is needed to clarify this hypothesis. In addition, the two predicted NLS motifs of *Dr*DDX41 existing beside the CC domain at the N-terminal region of the protein (1–190 amino acids) probably contribute to the nuclear localization of *Dr*DDX41 because the mutant protein without this N-terminal region no longer distributes in the nucleus. As another support, the N-terminal region (1–190 amino acids) with the two potential NLS motifs was localized in the nucleus. Moreover, the predicted NES motif (MDLLNKKMVSLDI) of *Dr*DDX41 included in the DEADc domain probably contributes to the nuclear exportation of *Dr*DDX41 because the protein with mutation in this motifs no longer transported from the nucleus into the cytoplasm, whether the cells were stimulated or not.

In general, the NF-κB, IFN, and chemokine-elicited immune reactions were orchestrated to play critical roles in host innate defense against microbial infection in vertebrates (2, 16). The observation that *Dr*DDX41 acting as an initiator for these reactions in zebrafish implies the particularly important function of DDX41 in innate anti-microbacteria immunity. This finding was finally supported by the performance of *Dr*DDX41 in the defense against the infection of *A. hydrophilia* and *E. tarda*. It can be anticipated that the finding of the wider participation of DDX41 in multiple innate immune reactions would provide a new perspective on the evolutionary history of the DDX41 family from fish to mammals as a whole.

## Ethics Statement

All animal work in this paper was conducted according to relevant national and international guidelines. All animal care and experimental procedures were approved by the Committee on Animal Care and Use and the Committee on the Ethic of Animal Experiments of Zhejiang University.

## Author Contributions

J-zS, J-xM, and WF conceived and designed the experiments. J-xM performed the experiments. J-xM and J-yL analyzed the data. L-xX and D-dF contributed reagents/materials/analysis tools. J-zS, J-xM, and J-yL wrote the manuscript. J-zS and A-fL reviewed the manuscript.

## Conflict of Interest Statement

The authors declare that they have no conflicts of interest with the contents of this article.

## Abbreviations

DDX41, DEAD (Asp-Glu-Ala-Asp)-box polypeptide 41; NLS, nuclear localization signal; NES, nuclear export signal; *Dr*DDX41, *Danio rerio* DDX41; *Dr*STING, *Danio rerio* STING; *Dr*STAT6, *Danio rerio* STAT6; *Dr*IL-6, *Danio rerio* IL-6; *Dr*TNFα, *Danio rerio* TNFα; *Dr*IFNφ1, *Danio rerio* IFNφ1; *Dr*Viperin, *Danio rerio* Viperin; *Dr*ISG15, *Danio rerio* ISG15; *Dr*CCL20, *Danio rerio* CCL20; PRRs, pattern-recognition receptors; TLRs, toll-like receptors; RLRs, RIG-I-like receptors; RIG-I, retinoic acid-inducible gene I; NOD, nucleotide oligomerization domain; NLRs, NOD-like receptors; CLRs, C-type lectin receptors; DAI, DNA-dependent activator of IFN-regulatory factors; AIM2, absent in melanoma 2; IFI16, IFNγ-inducible protein 16; cGAS, cyclic GMP-AMP synthase; dsDNAs, double-stranded DNAs; CDNs, cyclic dinucleotides; IKK, IκB kinase; TBK1, TANK-binding kinase 1; IRF-3/7, interferon regulatory factor 3/7; BTK, Bruton’s tyrosine kinase; STING, stimulator of IFN genes protein; STAT6, signal transduction and activator of transcription 6; MyD88, myeloid differentiation primary-response protein 88; TRIF, TIR-domain-containing adaptor protein inducing IFNβ; SOCS, suppressor of cytokine signaling; ISGs, interferon-stimulated genes; CC, coiled-coil; *Hs*DDX41, *Homo sapiens* DDX41; *Hs*ISG15, *Homo sapiens* ISG15; *Hs*IFNβ-Luc, *Homo sapiens* IFNβ-Luc; *Hs*IRF3-Luc, *Homo sapiens* IRF3-Luc; hpf, hours post fertilization; hpi, hours post infection; hpm, hours post microinjection; hps, hours post stimulation; RSR, relative survival rate; *A. h, Aeromonas hydrophilia*; *E. t, Edwardsiella tard*a; Q-RT-PCR, quantitative RT-PCR; Co-IP, coimmunoprecipitation; ns, not significant; *P. olivaceus, Paralichthys olivaceus*.
