# Supplementary material for: Identification of DEAD-Box RNA Helicase DDX41 as a Trafficking Protein That Involves in Multiple Innate Immune Signaling Pathways in a Zebrafish Model
Source: Front Immunol. 2018 Jun 11;9:1327. doi: 10.3389/fimmu.2018.01327 (PMC6005158; doi:10.3389/fimmu.2018.01327)
Supplement: Supplementary file 1 [file Data_Sheet_1.PDF]

Supplementary Material

Identification of DEAD-box RNA helicase DDX41 as a trafficking protein that involves in multiple innate immune signaling pathways in a zebrafish model

Running title: Involvement of DDX41 in multiple innate signaling pathways

Jun-xia Ma[1], Jiang-yuan Li[1], Dong-dong Fan[1], Wei Feng[1], Ai-fu Lin[1], Li-xin Xiang[1*], Jian-zhong Shao[1, 2*]

[1]College of Life Sciences, Key Laboratory for Cell and Gene Engineering of Zhejiang Province, Zhejiang University, Hangzhou, People’s Republic of China;
[2]Laboratory for Marine Biology and Biotechnology, Qingdao National Laboratory for Marine Science and Technology, Qingdao, People’s Republic of China

[*]**Correspondence:** Jian-zhong Shao, shaojz@zju.edu.cn; Li-xin Xiang, xianglx@zju.edu.cn.

Materials and methods

Electroporation for ZF4 cells transfection

Zebrafish ZF4 cells were cultured at 28 ℃ in DMEM-F12 cell culture medium (HyClone) with 110 μg/mL sodium pyruvate (CORNING) and 10% FBS (Bovogen). After being digested by 0.25% trypsin (ThermoFisher), the cell numbers were counted by using a hemocytometer. Approximately 2×10^6 cells were suspended in 200 μL of DMEM-F12 in a 0.4 cm electroporation cuvette and 20 μg of plasmid DNA was added (1). After electroporation (square wave pulses, 270 V, 25 ms, BioRad MicroPulser electroporator), the cells were suspended into 1 mL of DMEM-F12 medium and transferred onto two wells of 24-well plate containing coverslips. At 24 h post-transfection, ZF4 cells were fixed with 4% paraformaldehyde and analyzed by immunofluorescence staining as described in the main text.

(1) Fan, L., J. Moon, J. Crodian, and P. Collodi. Homologous recombination in zebrafish ES cells. *Transgenic Res.* (2006) 15 (1), 21-30. doi: 10.1007/s11248-005-3225-0

Supplemental Table 1. Primers for vector construction and Q-RT-PCR assay, and the MOs for knockdown assay.

Supplemental Figure 1

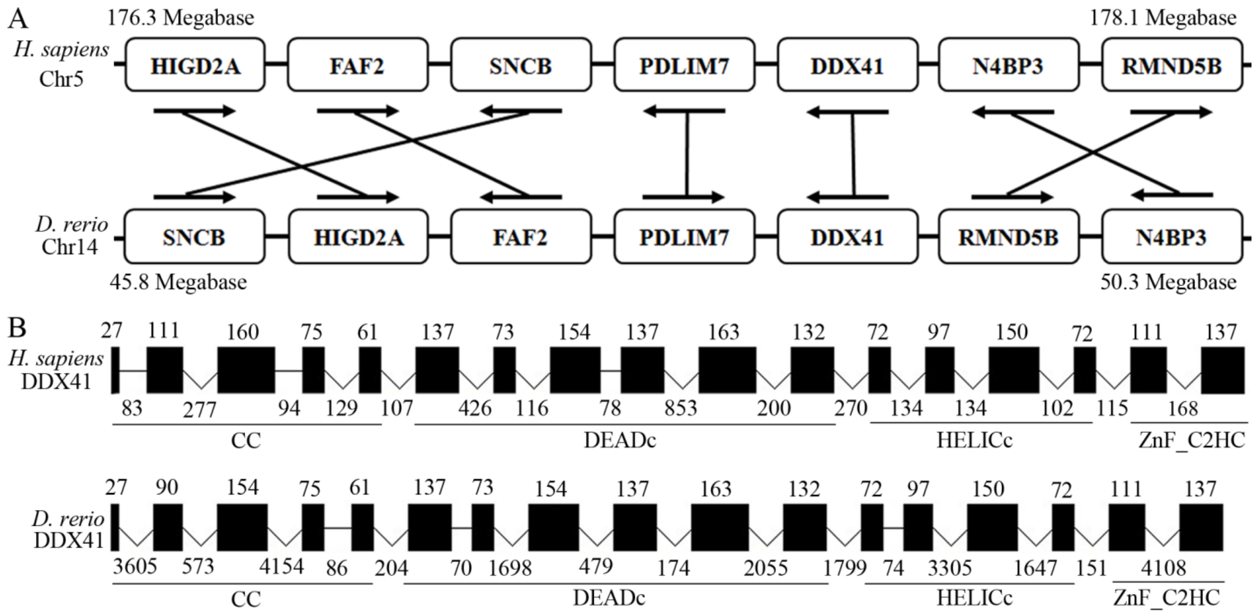

Supplemental Figure 1. Comparative analysis of the chromosomal location and organization of DDX41 genes between human and zebrafish. (**A**) Syntenic analysis of DDX41 gene and genes adjacent to DDX41 loci on human chromosome 5 (upper) and zebrafish chromosome 14 (lower). Arrows indicate the gene orientation. (**B**) Intron/exon structures of the *Dr*DDX41 gene compared with those of the *Hs*DDX41 gene. Exons and introns are indicated, respectively, with black boxes and adjacent lines. The length of each exon and intron is indicated by the number above and below, respectively. Schematic diagrams are included beneath the exon/intron organization cartoons to indicate the exons in terms of protein domains, including CC (coiled-coil), DEADc (central DEAD-box), HELICc (carboxy-terminal helicase), and ZnF_C2HC domains.

Supplemental Figure 2

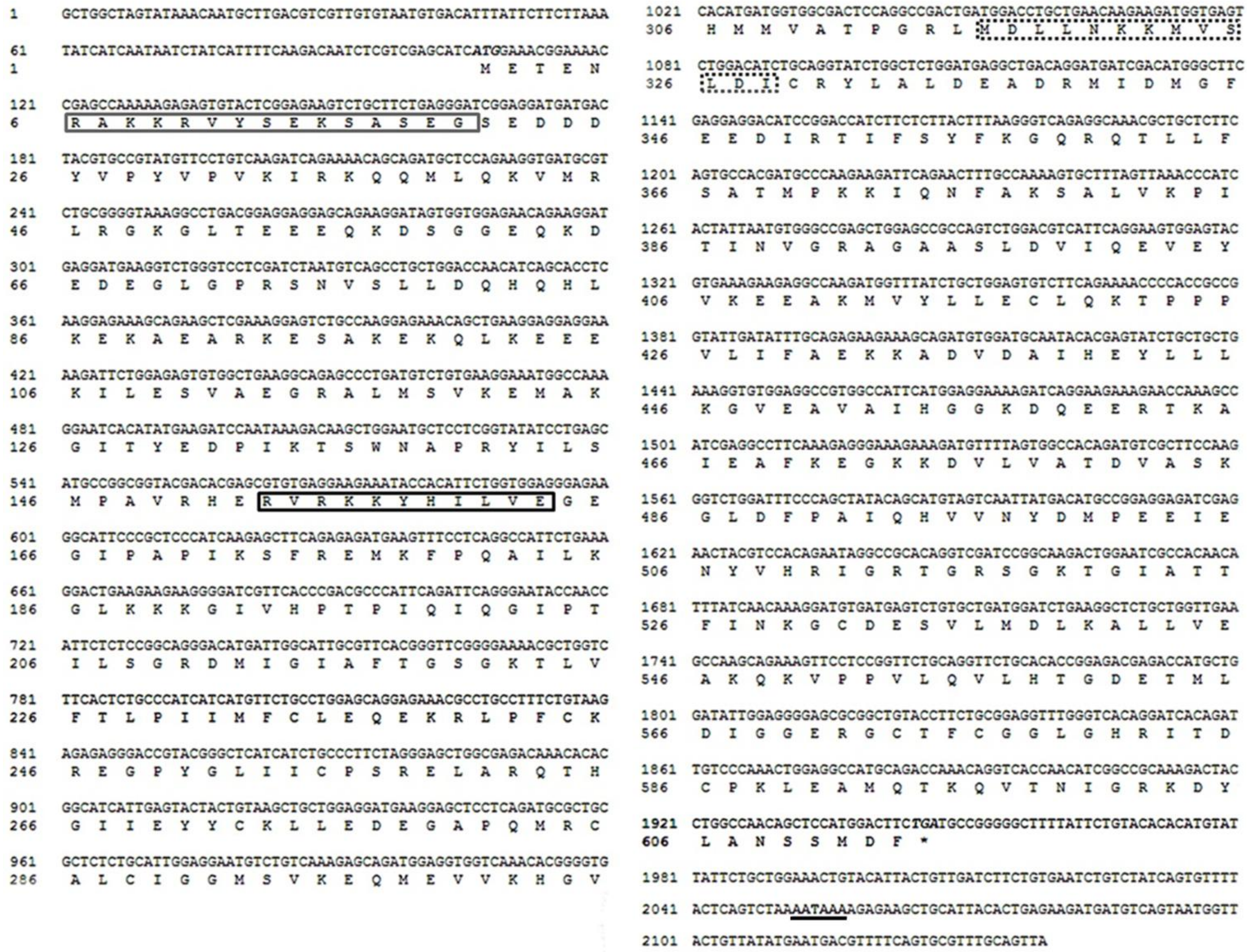

Supplemental Figure 2. Nucleotide and deduced amino acid sequence of the *Dr*DDX41 gene. The asterisk represents the stop codon, and two predicted NLS motifs and one NES motif are encircled with solid and dashed boxes respectively. In the 3′UTR, one poly(A) signals (AATAAA) is boldface and underlined.

Supplemental Figure 3

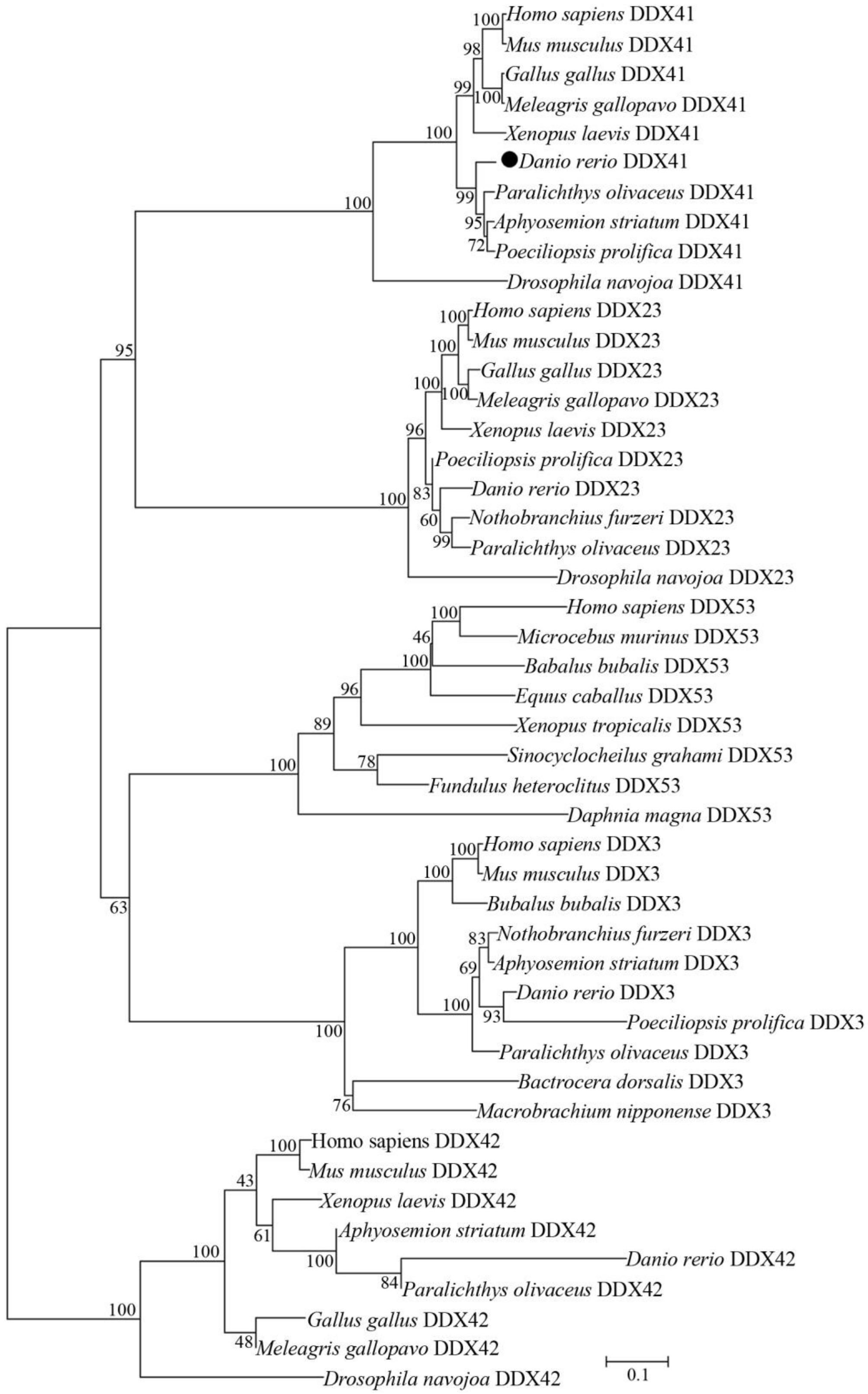

Supplemental Figure 3. Phylogenetic analysis of *Dr*DDX41. Phylogenetic tree was constructed by MEGA (version 4.1) using the neighbor-joining method. The tree shows the relationship of DDX41 among fish and other species. Other family members with the closest genetic relationship to DDX41, such as DDX3, DDX23, DDX42, and DDX53, were included in the analysis. The reliability of each node was estimated by bootstrapping with 1000 replications. The *numbers* shown at each node indicate the bootstrap values (%).The accession numbers for the sequences of DDX41 and other family members included in the phylogenetic analysis are as follows: *Homo sapiens* DDX41, NP 057306.2; *Mus musculus* DDX41, NP 598820.2; *Meleagris gallopavo* DDX41, XP 003210383.2; *Gallus gallus* DDX41, 1167503315; *Xenopus laevis* DDX41, 1069337040; *Danio rerio* DDX41, XP 017209912.1; *P. olivaceus*, DDX41, AIJ04334.1; *Poeciliopsis prolifica* DDX41, JAO78977.1; *Aphyosemion striatum* DDX41, SBP16344.1; *Drosophila navojoa* DDX41, JAC51753.1; *Homo sapiens* DDX23, NP 004809.2; *Mus musculus* DDX23, NP 001074450.1; *Gallus gallus* DDX23, 971447398; *Meleagris gallopavo* DDX23, 971447398; *Xenopus laevis* DDX23, XP 018105480.1; *Danio rerio* DDX23, NP 956176.1; *P. olivaceus* DDX23, 1143394425; *Poeciliopsis prolifica* DDX23, JAO16269.1; *Nothobranchius furzeri* DDX23, XP 015823869.1; *Drosophila navojoa* DDX23, 1062659759; *Homo sapiens* DDX53, NP 874358.2; *Microcebus murinus* DDX53, 829865379; *Bubalus bubalis* DDX53, 594032079; *Equus caballus* DDX53, NP_001244012.1; *Xenopus tropicalis* DDX53, AAI21889.1; *Sinocyclocheilus grahami* DDX53, 1020444626; *Fundulus heteroclitus* DDX53, 1012787768; *Daphnia magna* DDX53, JAM94845.1; *Homo sapiens* DDX3, AAC34298.1; *Mus musculus* DDX3, NP 034158.1; *Bubalus bubalis* DDX3, 726973479; *Poeciliopsis prolifica* DDX3, JAO15999.1; *Danio rerio* DDX3, AAI33163.1; *P. olivaceus* DDX3, AKS43550.1; *Nothobranchius furzeri* DDX3, SBP51991.1; *Aphyosemion striatum* DDX3, SBP03189.1; *Macrobrachium nipponense* DDX3, ADB28896.1; *Bactrocera dorsalis* DDX3, 618055618; *Homo sapiens* DDX42, NP 987095.1; *Mus musculus* DDX42, EDL34279.1; *Danio rerio* DDX42, NP 001032894.2; *Meleagris gallopavo* DDX42, XP 003213086.1; *Gallus gallus* DDX42, NP 001026097.1; *Xenopus laevis* DDX42, NP 001080569.1; *P. olivaceus* DDX42, 1143391314; *Aphyosemion striatum* DDX42, 1074586325; *Drosophila navojoa* DDX42, 1062665231.

Supplemental Figure 4

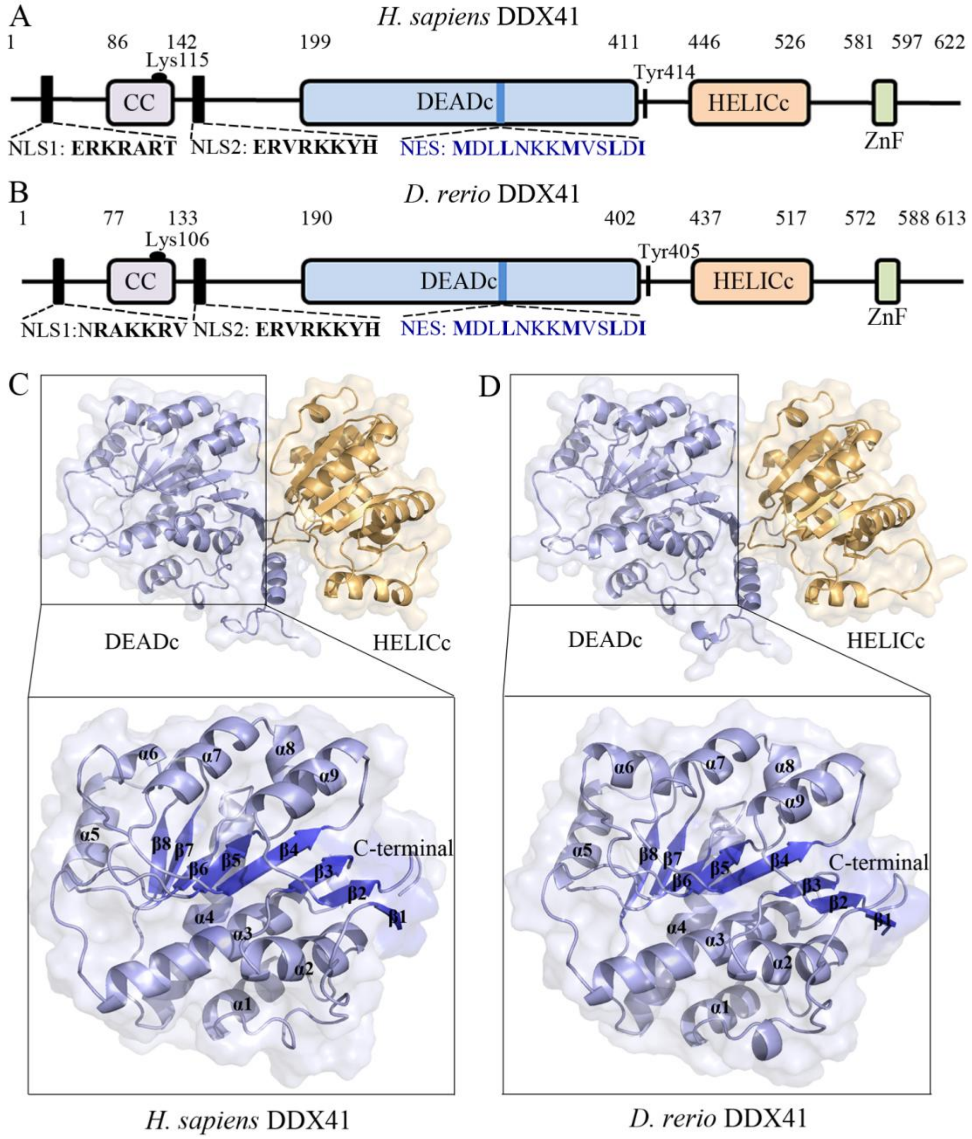

Supplemental Figure 4. Schematic diagram of full-length protein and tertiary structure of the RecA-like DEADc domain of human and zebrafish DDX41. Zebrafish DDX41 (*Dr*DDX41) (**A**) exhibits an overall structural conservation to human counterparts (**B**) as evidenced by the similar coiled-coil (CC, light purple), DEADc (light blue), HELICc (light orange), and ZnF_C2HC (ZnF, light green) domains. The DDX41 protein was predicted to have two NLS motifs beside the coiled-coil domain and one ubiquitination site in it, as well as a tyrosine phosphorylation site beside the right of the DEADc domain and an NES motif in it, all of which are indicated as in the diagram. (**C** and **D**) The upper images show the structure of DEADc (colored in light blue) and HELICc (colored in light orange) domain of human (**C**, PDB ID: 2P6N) and zebrafish (**D**). The lower images show the detailed views of the RecA-like DEADc domain consisting of one core β-sheets (colored in tv_blue) surrounded by 9 α-helixes and the similar C-terminal region. The

3D structures were modeled by SWISS-MODEL and I-TASSER and then decorated by PyMOL molecular graphic software version 1.3.

Supplemental Figure 5

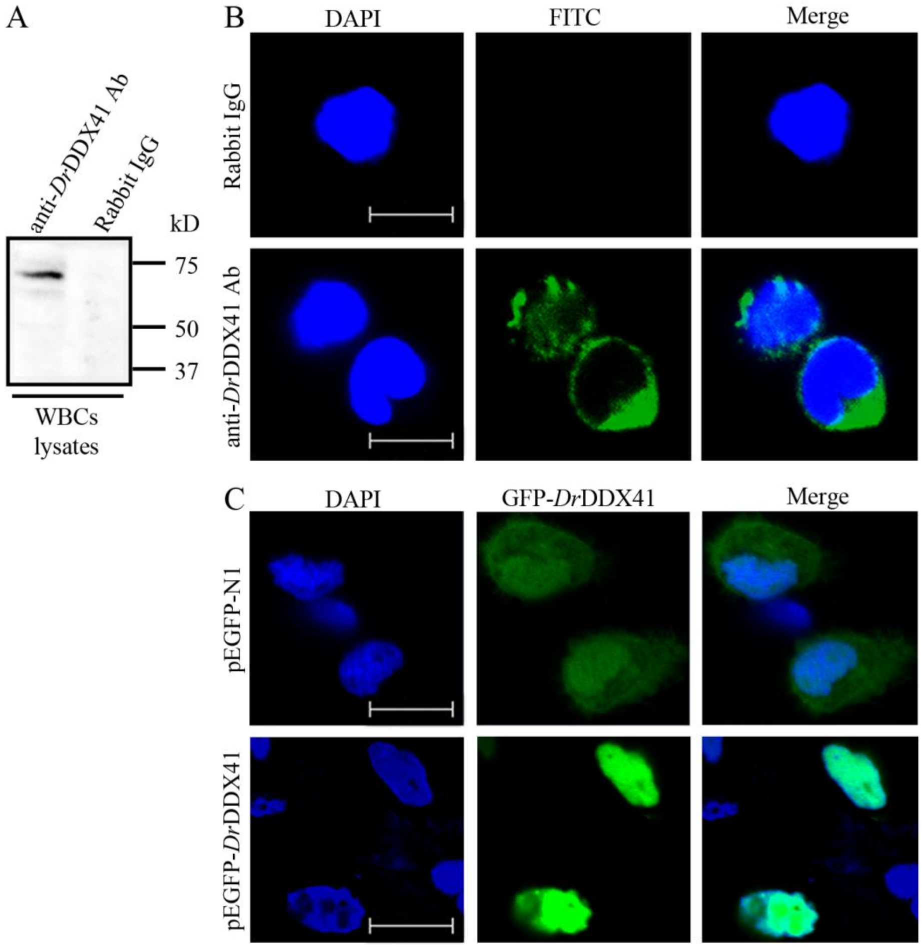

Supplemental Figure 5. Subcellular localization analysis of *Dr*DDX41 in zebrafish WBCs and ZF4 cells. (A) Western blot shows that the rabbit anti-*Dr*DDX41 Ab can specifically bind to the corresponding endogenous target proteins in the whole cell lysates of the WBCs sorted from the peritoneal blood, kidney, and spleen. (B) Confocal microscopy image of WBCs stained with rabbit anti-*Dr*DDX41 Ab, showing that the *Dr*DDX41 protein partially distributed in nucleus and cytoplasm in the natural WBCs. WBCs stained with a non-related rabbit IgG was used as negative control. (C) Representative images of transfected ZF4 cells with pEGFP-N1 and pEGFP-*Dr*DDX41. Nuclei were stained with the DNA-intercalating dye DAPI. Scale bars represent 5 μm. Images were captured under a laser scanning confocal microscope (Zeiss LSM-710; original magnification, 630×).

Supplemental Figure 6

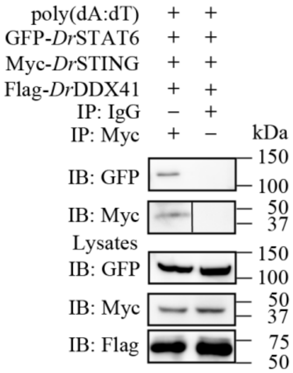

Supplemental Figure 6. *Dr*STING interacts with *Dr*STAT6. HEK293T cells were transfected with GFP-*Dr*STAT6, Myc-*Dr*STING and Flag-*Dr*DDX41 for 24 hour and then stimulated with poly(dA:dT) for 6h. Cell lysates were immunoprecipitated with anti-Myc antibody (Myc) or control mouse IgG (IgG), and analyzed by western blot using anti-GFP and anti-Myc antibodies. Expression of the transfected plasmids was analyzed with anti-GFP, anti-Myc, and anti-Flag antibodies in the whole cell lysates.
